# Hybrid transcriptome sequencing approach improved assembly and gene annotation in *Cynara cardunculus* (L.)

**DOI:** 10.1186/s12864-020-6670-5

**Published:** 2020-08-21

**Authors:** Giuseppe D. Puglia, Andrey D. Prjibelski, Domenico Vitale, Elena Bushmanova, Karl J. Schmid, Salvatore A. Raccuia

**Affiliations:** 1 Institute for Plant Breeding, Seed Science and Population Genetics, University of Hohenheim, Fruwirthstrasse 21, 70599 Stuttgart, Germany; 2 Consiglio Nazionale delle Ricerche, Istituto per i Sistemi Agricoli e Forestali del Mediterraneo (CNR-ISAFOM) U.O.S. Catania, Via Empedocle, 58, 95128 Catania, Italy; 3 Center for Algorithmic Biotechnology, Institute of Translational Biomedicine, St. Petersburg State University, St. Petersburg, Russia

**Keywords:** Hybrid-seq, RNA-seq, de novo transcriptome assembly, *Cynara cardunculus*, Inflorescence development, Alternatively spliced isoforms, Isoform detection, Gene annotation

## Abstract

**Background:**

The investigation of transcriptome profiles using short reads in non-model organisms, which lack of well-annotated genomes, is limited by partial gene reconstruction and isoform detection. In contrast, long-reads sequencing techniques revealed their potential to generate complete transcript assemblies even when a reference genome is lacking. *Cynara cardunculus* var. *altilis* (DC) (cultivated cardoon) is a perennial hardy crop adapted to dry environments with many industrial and nutraceutical applications due to the richness of secondary metabolites mostly produced in flower heads. The investigation of this species benefited from the recent release of a draft genome, but the transcriptome profile during the capitula formation still remains unexplored. In the present study we show a transcriptome analysis of vegetative and inflorescence organs of cultivated cardoon through a novel hybrid RNA-seq assembly approach utilizing both long and short RNA-seq reads.

**Results:**

The inclusion of a single Nanopore flow-cell output in a hybrid sequencing approach determined an increase of 15% complete assembled genes and 18% transcript isoforms respect to short reads alone. Among 25,463 assembled unigenes, we identified 578 new genes and updated 13,039 gene models, 11,169 of which were alternatively spliced isoforms. During capitulum development, 3424 genes were differentially expressed and approximately two-thirds were identified as transcription factors including bHLH, MYB, NAC, C2H2 and MADS-box which were highly expressed especially after capitulum opening. We also show the expression dynamics of key genes involved in the production of valuable secondary metabolites of which capitulum is rich such as phenylpropanoids, flavonoids and sesquiterpene lactones. Most of their biosynthetic genes were strongly transcribed in the flower heads with alternative isoforms exhibiting differentially expression levels across the tissues.

**Conclusions:**

This novel hybrid sequencing approach allowed to improve the transcriptome assembly, to update more than half of annotated genes and to identify many novel genes and different alternatively spliced isoforms. This study provides new insights on the flowering cycle in an Asteraceae plant, a valuable resource for plant biology and breeding in Cynara and an effective method for improving gene annotation.

## Background

The botanical species *Cynara cardunculus* L. includes globe artichoke (subsp. *scolymus* (L.) Hegi), cultivated cardoon (var. *altilis* DC.) and the wild cardoon (var. *sylvestris* (Lamk) Fiori). Both *scolymus* and *altilis* have been traditionally used for centuries as vegetables in South Europe for the immature capitulum inflorescence and for the edible stalks, respectively. The cultivated cardoon is recently drawing increasing research interest for its potential as an industrial crop for biomass, grain and oil production especially in dry and marginal lands [1–3] and, only more recently, as source of bioactive molecules for human consumption [4–6]. In particular, the capitulum contains antioxidant compounds of substantial quantity and quality, and only few studies investigated such compounds in cultivated cardoon so far [7, 8]. In contrast, more work has been done on phenolic acid composition in globe artichoke flower heads [9–11], which shows significant variation in quality and quantity, depending on the harvesting stage [12]. These phenol compounds are mostly mono- and di-caffeoylquinic acids (CQAs) with a relevant role as structural components of plant cell walls during flowering [13], and flavonoids, mostly represented by apigenin, luteonin and their conjugates; they protect the plant from solar irradiation acting as reactive oxygen species (ROS) scavenger [14]. Both phenolic acids and flavonoids have been related to a positive effect on cancer prevention [15] and to reduce the invasiveness of human breast cancer cells line, triggering apoptosis [16]. Notwithstanding the relevance of polyphenol pathways, their expression dynamics during the capitulum development has not been completely investigated so far in *C. cardunculus* taxa. Recently, the isolation of some genes involved in the phenylpropanoid biosynthesis [17–19] and in the regulation of flavonoid pathway [20, 21] provided insights on the molecular mechanisms controlling polyphenol accumulation in this plant.

Among secondary plant metabolites, the sesquiterpene lactones exhibit widely known allochemical properties [22, 23]. In *C. cardunculus* cynaropicrin, grosheimin and its derivatives were identified [24, 25], and their use as pharmaceutical agents were proposed due to their potential for the treatment of cardiovascular disease and cancer [26, 27]. The recent genetic mapping of key genes in the STL pathway for the production of cynaropicrin [28] showed a correlation between germacrene synthase A (GAS) expression and cynaropicrin content that supports a role of this enzyme in the corresponding biosynthetic pathway.

Realizing the importance of *C. cardunculus* as crop plant with so many relevant applications, lately a reference genome for globe artichoke through a pipeline for low coverage (< 1×) whole genome-sequencing segregating population was generated, which provided an initial description of genome organization and gene content [29, 30]. To the best of our knowledge, only one study analysed the transcriptome of this species [31] focusing on the identification of SNPs and microRNA targets without evaluating the expression dynamics, though. Nevertheless, a comprehensive investigation on the expression dynamics is required to understand the molecular regulation of developmental processes and the production of valuable compounds present in the flower heads.

Flowering is a crucial developmental step in the higher plant life cycle for their reproductive success and a considerable amount of valuable compounds are produced during this step [32]. The molecular regulation of flower development has been studied at the transcriptome level through RNA-seq in other plant species, particularly in model organisms like *Arabidopsis thaliana, Glycine max* and *Medicago truncatula* [33–35] and for some non-model organism like pomegranate and chickpea [36, 37]. Most differentially expressed genes (DEGs) identified in these studies encoded for transcription factors indicating the importance of regulatory networks in flower development. Although RNA-Seq is a valuable approach for interpreting the functional elements of the genome, its application to non-model organisms is frequently limited by the absence of a reference genome or comprehensive annotation [38, 39]. Moreover, most current transcriptome studies are performed with short read-sequencing (SR-seq) which retrieves a large number of transcripts, but with potential limitations, especially in non-model organisms. These limitations include the generation of chimaera, fragmented genes and reduced isoform discovery [40, 41]. Recently, long read sequencing (LR-seq) that covers transcripts in their full length has been exploited successfully in plants to describe transcriptome complexity [42, 43] or was used in targeted sequencing [44]. Among LR-seq platforms, ONT-Nanopore technology with a portable small size and low equipment costs represents an affordable resource for LR-seq [45], but its higher error rate respect to SR-seq and the requirement of high coverage makes the accurate de novo assembly challenging [46]. Genome hybrid sequencing (LR-seq + SR-seq) has emerged as a novel approach to overcome the limitations of the two sequencing approaches, but currently is mainly applied to the assembly of model organisms genome [47]. Although a tool for hybrid RNA-seq assembly was recently proposed [48], its application remained limited to a proof-of-concept use and showed no significant improvement over SR-seq assembly. To perform de novo assembly of LR-seq and SR-seq simultaneously in *C. cardunculus* with a limited genome annotation, we adapted the recently developed rnaSPAdes assembler [49] by modifying the isoform reconstruction algorithm implemented in exSPAnder framework [50]. The resulting hybrid RNA-seq assembly showed significant improvement over SR-seq assembly.

In this study, we analysed the global gene expression dynamics during flowering development in *C. cardunculus* var. *altilis* using short and long reads to generate an improved de novo transcriptome assembly providing a higher quality genome annotation, gene models representation and an enhanced isoforms detection.

## Results

### RNA sequencing

**Fig. 1:**
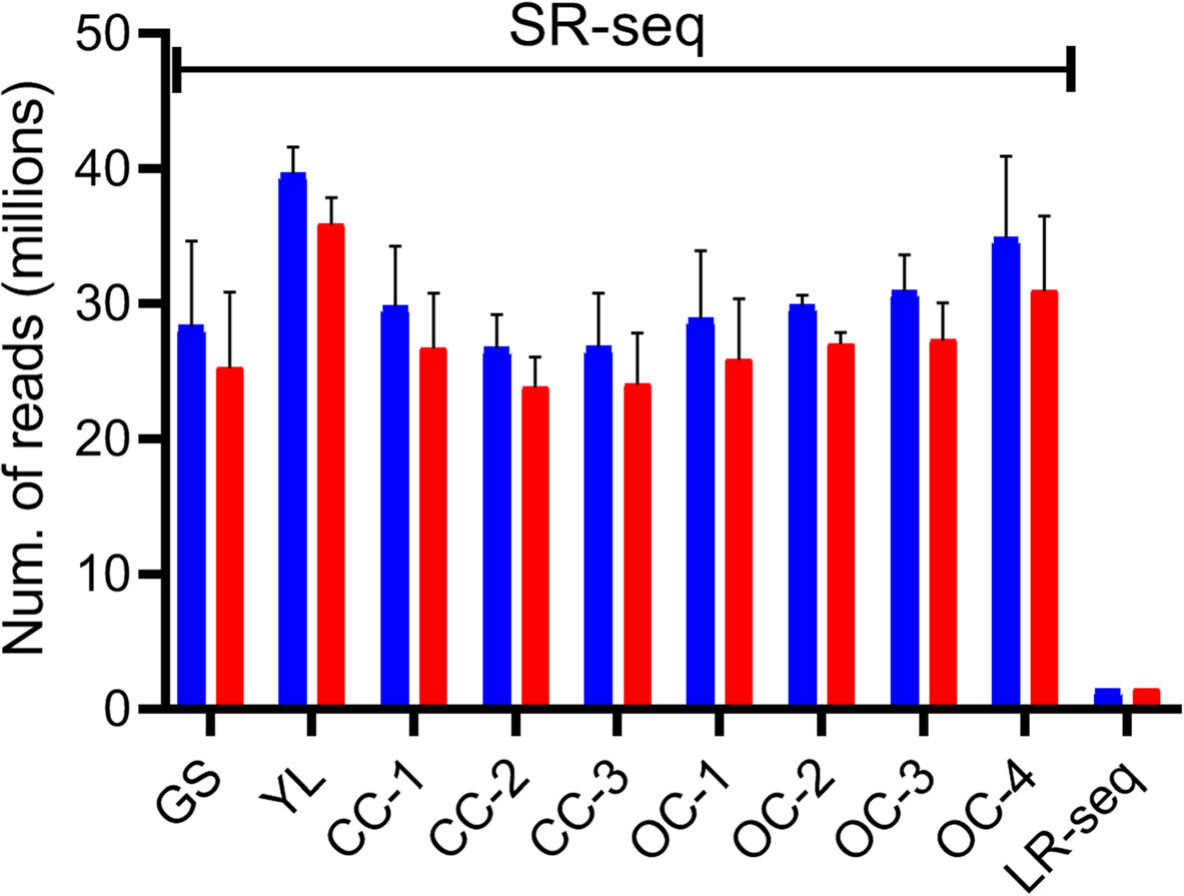
Numbers of SR-seq and LR-seq reads, and length distribution of contigs and transcripts. **a** Raw (blue bars) and trimmed (red bars) read counts (millions) average for each sample for SR-seq and LR-seq (from a pool of samples). Thin bars the standard deviation (SD) of read counts between replicates

We obtained a total of 1,023,768,646 read pairs from 34 samples (libraries) for Illumina with a total read coverage of 280x (average of 95x in each sample) with a mean Q always above 33; while from Nanopore sequencing, 1,445,444 reads were obtained from a 48-h run (Table S2 and Fig. 1) with a read coverage of 2x. Nanopore reads were significantly longer in length (on average 665 bp), 93% of which were recognized as a full-length transcript. Using minimap2, 86% of reads from LR-seq were mapped to *C. cardunculus* reference genome. Mapping rate for SR-seq using STAR was 89% of the total reads.

### Hybrid RNA de novo assembly

**Fig. 2:**
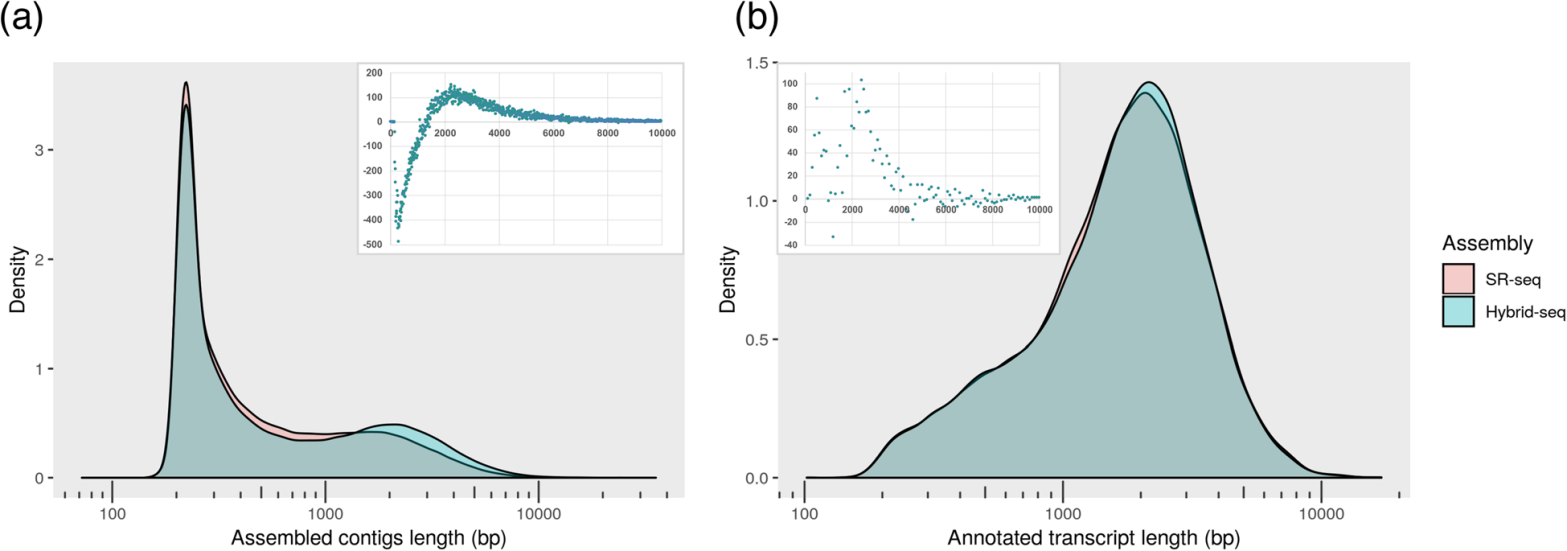
Comparison of sequence length distribution of SR-seq (light red) versus hybrid-seq assembly (light green) for contigs (**a**) and for transcript sequences (**b**) obtained from the PASA software. Insets in the figures show fragment length delta of hybrid-seq assembly respect to SR-seq with contigs (**a**) or transcripts (**b**) length in the x-axes and number of occurred fragments in the y-axes

The reads from both sequencing platforms were combined to perform a hybrid transcriptome assembly to be compared to the assembly using only short reads. The distribution length of the contigs and the transcripts obtained from the two sequencing approaches resulted in slight differences (Fig. 2a and b). For both sequencing approaches the highest peak was at 220 bp with a higher proportion of assembled contigs with SR-seq reads (500 more respect to hybrid-seq), while hybrid-seq showed a higher assembled contigs proportion between 1360 bp and 7260 bp with the relative highest peak at 2390 bp with 145 more respect to SR-seq (Fig. 2a). As for transcript length distribution, albeit with a lesser extent respect to contigs, hybrid-seq showed a higher proportion of longer transcripts, among which the most numerous difference length was at 2400 bp with 103 more longer transcripts respect to SR-seq (Fig. 2b). In total, the transcriptome assembly obtained with hybrid-seq reads produced 7495 more transcripts (1.56%) than SR-seq. Among the transcripts generated with hybrid-seq, 19,043 (10.83%) were longer (≥ 500 bp) and, according to rnaQuast analysis, 1952 more transcripts (15.09%) showed a 95%-assembled gene assembly respect to SR-seq (Table 1). Also TransRate software estimated an increase in the number of 95%-covered genes (6% more), N50 (30% higher), optimal score and a significant decrease in the number of segmented contigs (68% fewer) for assemblies generated with hybrid-seq respect to SR-seq. Likewise, BUSCO analysis showed that with the hybrid approach 92 more complete universal single-copy orthologs were restored for its plant database reaching a total of 1330 (95% of the entire database).

**Table 1:** Summary statistics for quality assessment of the assemblies obtained with SR-seq and hybrid-seq

	SR-seq	Hybrid-seq
No. of transcripts	480,403	487,898
Transcripts > 500 bp	175,817	194,860
*rnaQUAST metrics*		
Aligned transcripts	230,373	238,818
50%-assembled genes	19,355	19,486
95%-assembled genes	12,932	14,884
*TransRate metrics*		
95% covered genes	13,038	13,800
Segmented contigs	56,825	18,422
N50	1803	2342
Optimal score	0.11	0.26
*BUSCO metrics*		
Complete BUSCOs	1238	1330

### PASA alignment and annotation

**Fig. 3:**
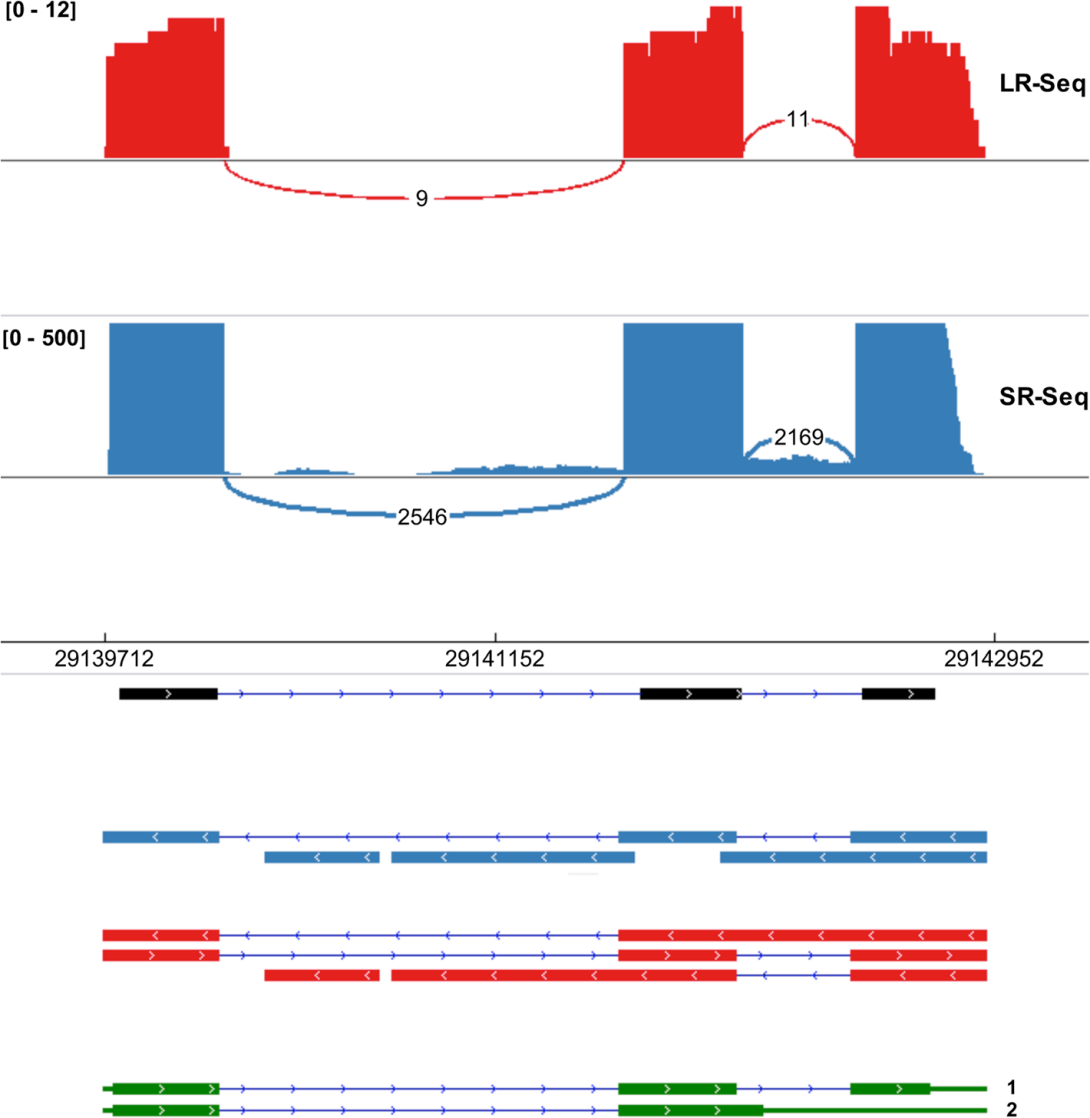
Updated annotation of gene Ccrd_v2_09297_g05, flavanone 3-dioxygenase (F3H), using both LR-seq and SR-seq with different gene model alternative splicing isoforms (Sashimi plot). Existing annotation (black bars) is compared with SR-seq (blue bars) and hybrid-seq (red bars) assemblies and with the validated PASA updated annotation (green bars) including 2 different isoforms: Ccrd_v2_09297_g05-mRNA-1 (1) and Ccrd_v2_09297_g05-mRNA-1-1.5b860155 (2). Intronic regions are shown by lines and UTRs by thinner green bars

In this study 502 and 578 unannotated genes were identified, respectively for SR-seq and hybrid-seq approaches, which were not present in the current globe artichoke genome annotation (Table 2). Moreover, the hybrid-seq approach allowed to detect 18.6% more of alternative isoforms compared to the assembly made up by short-reads. Also, the composition of alternative splicing types was slightly different in the two assemblies detected by PASA (Table S3). In fact, in the hybrid-seq assembly, more gene counts (180) were involved in alternative splicing characterized by higher alternate exchanges and intron retention and a lower exon skipping events. Most gene model updates accounted for alternative gene structure and for UTR modifications at 5′ and 3′ ends of genes. As an example, for the gene Ccrd_v2_09297_g05, encoding for flavanone 3-dioxygenase (F3H), in the existing annotation a single transcript is present with three exons and two introns (Fig. 3). The SR-seq data were fragmented and from the combination of multiple contigs only one complete transcript, Ccrd_v2_09297_g05-mRNA-1 (isoform 1), was reconstructed with an expanded exon and larger both 5′ and 3′-UTRs. However, with the use of hybrid-seq, full-length transcripts were retrieved which enabled to identify a new alternatively spliced isoform, Ccrd_v2_09297_g05-mRNA-1-1.5b860155 (isoform 2), differing for introns and exons number and 3′-UTR length (Fig. 3).

**Table 2:** Comparison of annotations of SR-seq and hybrid-seq assemblies refined and updated by PASA software. Existing artichoke genome annotation was used as reference

	SR-seq	Hybrid-seq
Newly annotated genes	502	578
Entirely new	302	551
Split genes	200	227
Updated genes	13,796	12,924
Alternative gene structure	8107	7769
UTR alternation	4838	4332
Protein modification	939	870
Gene merging	114	115
Alternatively spliced isoforms	8945	10,613
Proteins modified	9341	8960

### In silico functional annotation

**Fig. 4:**
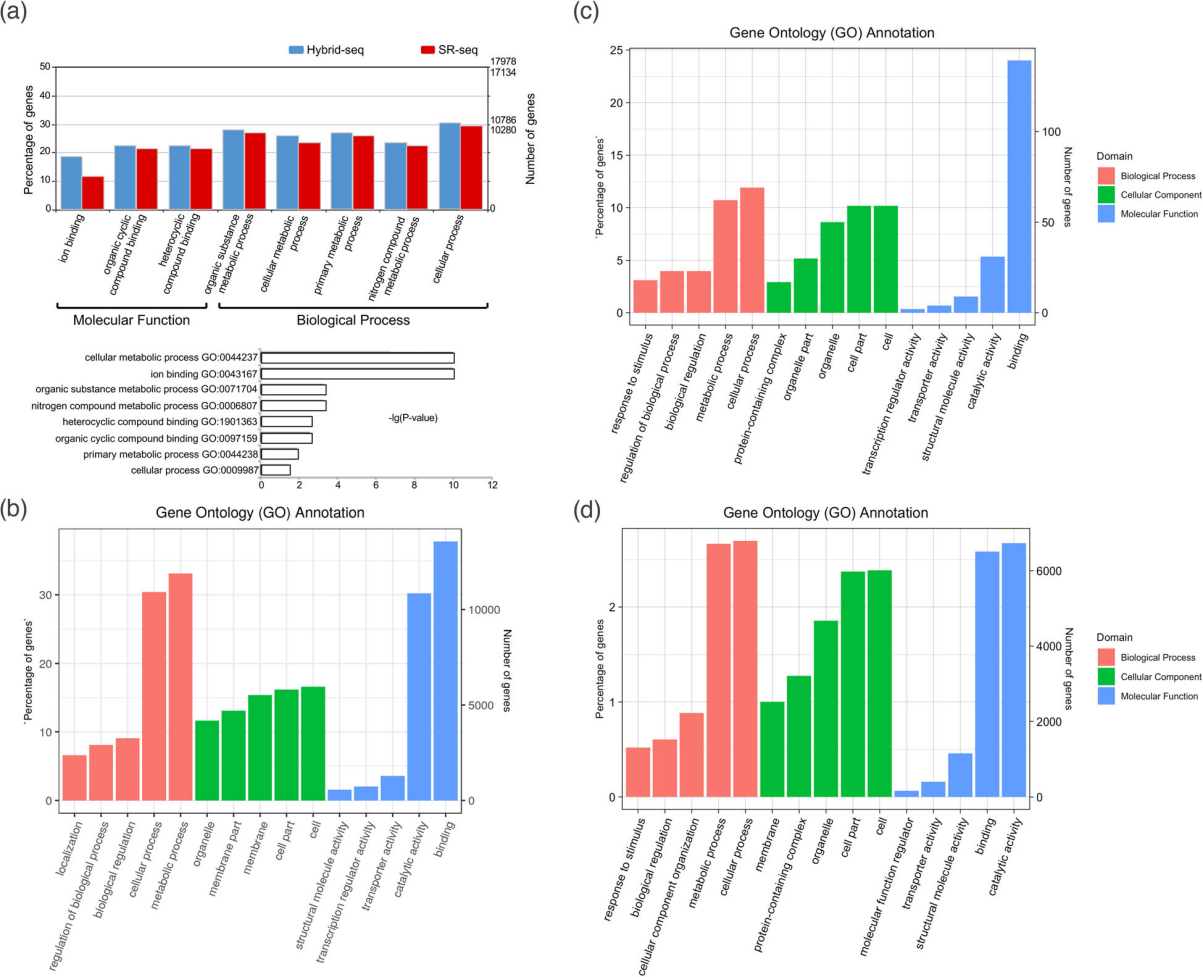
Gene Ontology (GO) annotation of the transcriptome data. **a** Significantly different GO terms (*p* value ≤0.05) obtained from the comparison between hybrid-seq and SR-seq with WEGO web application. Transcriptome annotation classification reporting the top five most abundant GO terms for Biological Process, Molecular Function and Cellular Component functional groups for hybrid-seq assembly (**b**) novel gene models annotated in this study (**c**) and unaligned contigs to the reference genome (**d**)

The in silico functional annotation of hybrid-seq identified 1688 more predicted genes and 1317 annotated genes respect to SR-seq assembly annotation (Table S4). As a consequence, for hybrid assembly 2249 more GO terms were retrieved respect to SR-seq. Between the two transcriptome assemblies annotations, the most significant difference (*p* value ≤0.05) in GO terms composition was found for molecular function (3 subcategories) and biological process (5 subcategories) (Fig. 4a). Within the molecular function, most different GO terms amount accounted for ‘ion binding’, while for biological process the ‘cellular metabolic process’ was the most different subcategory.

We used the hybrid-seq assembly for all analyses described here following. With the GO annotation analysis, we found 53 functional groups (Figure S2a), including biological process (30 subcategories), cellular component (13 subcategories) and molecular function (10 subcategories), which five most abundant GO terms are reported in Fig. 4a. For biological process, ‘cellular process’ and ‘metabolic process’ were dominant terms, while for molecular function ‘catalytic activity’ and ‘binding’ were the major subcategories. For cellular component, the identified GO terms were more evenly spread across the subcategories with ‘cell’, ‘cell part’ and ‘membrane’ accounting for the most numerous ones. As regards to the 578 newly identified genes, no BLAST hit was obtained for 79 sequences, while 306 GO terms corresponding to 32 functional groups (Figure S3), including biological process (16 subcategories), molecular function (7 subcategories) and cellular component (8 subcategories) were found for the 499 functionally annotated sequences, which five most abundant GO terms are reported in Fig. 4c. The relative enrichment analysis identified three and seven most represented GO terms for molecular function and biological process, respectively. The term ‘nucleic acid binding’ was most abundant by the number of genes, and GO groups related to RNA regulation were the most frequent (Figure S3). The 247,238 unaligned contigs resulted in 18,042 BLASTX hits with *Camelia sinensis*, *A. thaliana* and *Glycine soja* among the most represented hit species (Figure S4). As regards to the GO analysis, 11,691 sequences were in silico functionally annotated corresponding to 56 GO functional groups which showed a similar composition to hybrid-seq and novel gene models assemblies (Fig. 4d and Figure S5).

### Differential expression analysis

The PCA analysis carried out on the whole-gene expression data set showed the distinctness of the vegetative tissues (GS and YL) from the reproductive ones (CC and OC) (Figure S6). In addition, among the inflorescence tissues, the closed capitulum stages (CC-1 – CC-3) displayed a tight clustering; likewise, the open inflorescence stages (OC-1 – OC-4) clustered together. This indicates the presence of transcriptional signatures that can differentiate among distinct tissue types and within the inflorescence developmental stages. The analysis of the differentially expressed genes (DEG) across all the samples resulted in 2986 sequences. Verification of expression profiles of seven selected genes in the sampled tissues by qRT-PCR, showed a good correlation (R^2^ = 0.72) with RNA-seq (Figures S7 and S8), supporting the reliability of our dataset. From the examination of the expression dynamics, vegetative and reproductive tissues showed a very different profile indicating the presence of different transcriptional programmes regulating their development (Figure S9). The obtained DEGs set was used to perform the GO enrichment analysis, which detected 127 significantly enriched terms grouped into three main categories (biological process, molecular function and cellular component) (Figure S10 and S11). Among GO terms, the most abundant were ‘binding’ (48%; GO:0005488) and ‘catalytic activity’ (47%; GO:0003824) for molecular function, while for biological process were ‘metabolic process’ (44%; GO:0008152) and ‘cellular process’ (41%; GO:0009987), and ‘cell part’ (27%; GO:0044464) and ‘cell’ (27%; GO:0005623) were the most numerous for cellular component.

**Fig. 5:**
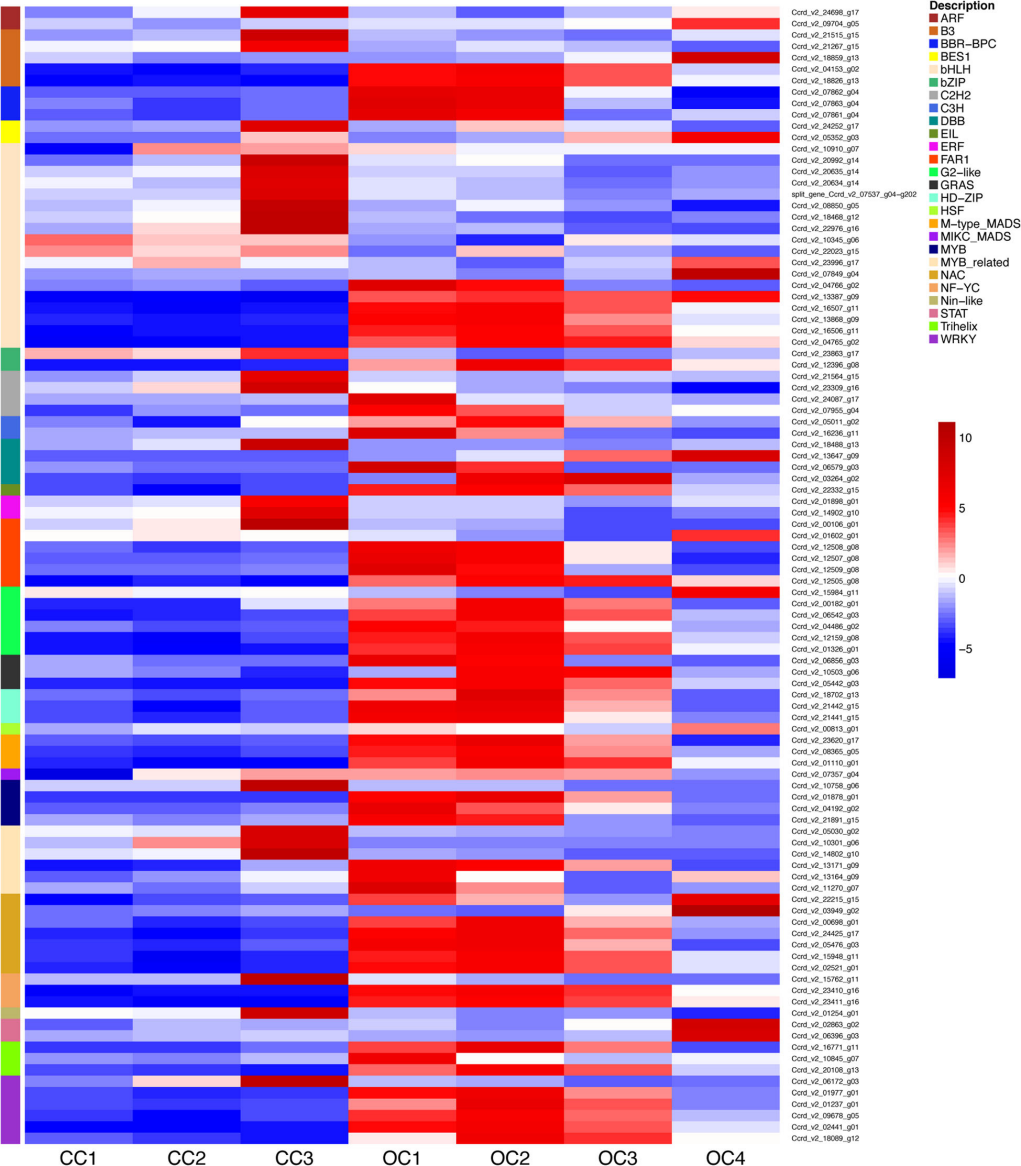
Transcriptional dynamics during inflorescence development of top 100 differentially expressed TFs grouped for each belonging family. The colour scale represents the log_2_-transformed TPM value

Regulation of transcription is known to play a pivotal role in the flowering development. Among the 3424 differentially expressed genes across capitulum formation samples (Figure S12), 2203 genes accounted for transcription factors (Fig. 5, Figures S13 and S14). In general, we observed different expression dynamics between the closed and the opened capitulum with a more pronounced up-regulation of TF gene expression in the open capitulum stages. The TF family with the highest number of representatives were bHLH (368) which exhibited the highest proportion of up-regulated genes at OC-2 stage (38%) while CC-1 was the stage with the largest bHLHs down-regulation (52.4%). The second most abundant TF family was MYB/MYB-related (221), which were mostly (46.6% at CC-1) down-regulated in the first steps of inflorescence formation and showed the largest number of up-regulated genes (36.2%) at CC-3 stage. While NAC family accounted for 149 genes mostly over-expressed in the final stages of inflorescence development and in a lesser extent in middle of this process. C2H2 (103 genes) TF family was up-regulated during later stages of the closed capitulum and early stages of the opening inflorescence. While WRKY genes (94) were mostly expressed at the start of capitulum opening and throughout the OC stages. MADS (M-type + MIKC) TF family, instead, accounted for 81 members that were mostly (63%) down-regulated at early inflorescence developmental stages, while at later stages 45% of genes were up-regulated.

### Phenylpropanoids and flavonoids biosynthetic pathways

**Fig. 6:**
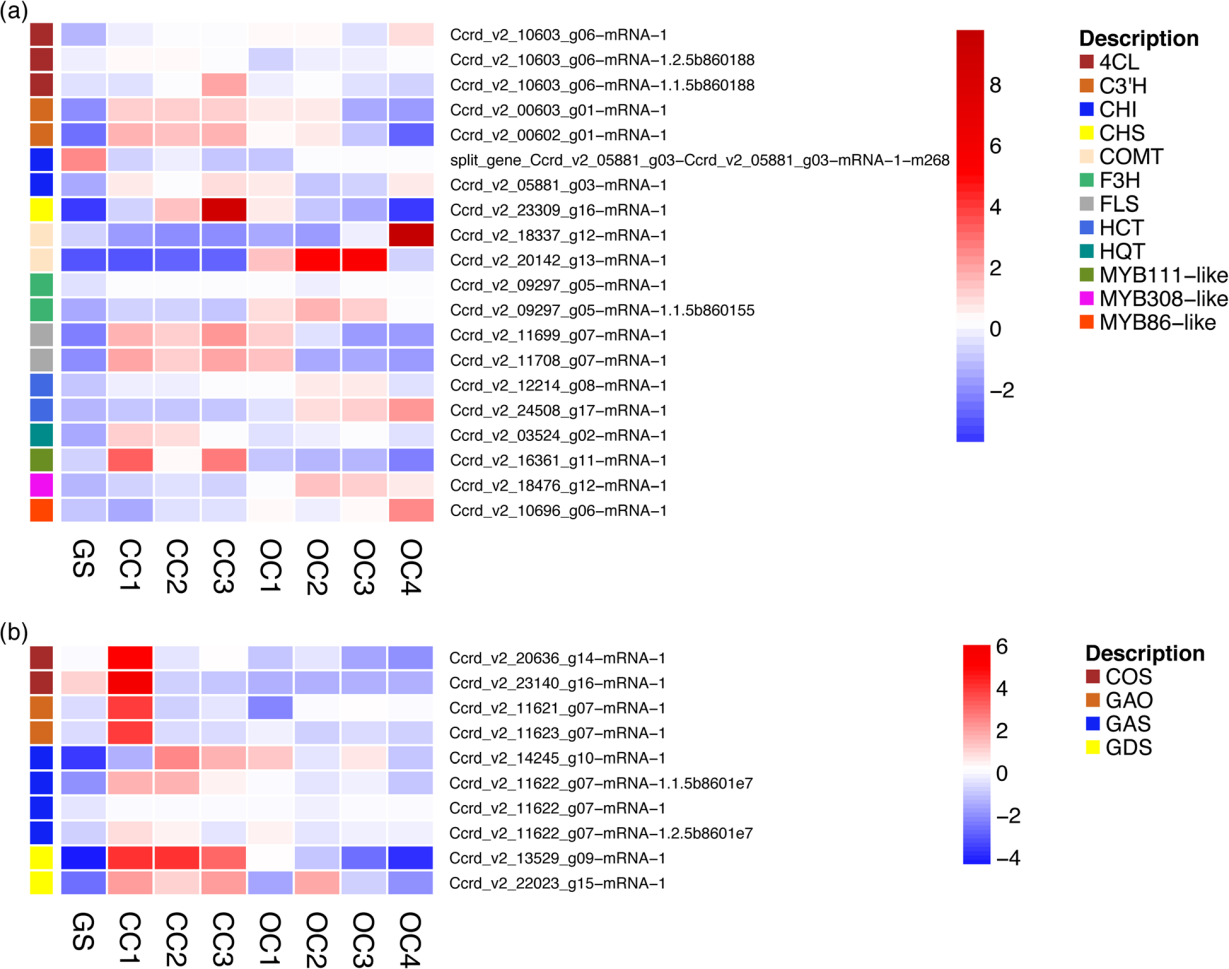
Expression heat maps in germinating seedling (GS) and inflorescence developmental stages (from CC1 to OC4) of genes related to the biosynthesis of phenylpropanoids and flavonoids (**a**) and sesquiterpene lactones (**b**). *4CL* 4-coumarate-CoA ligase, *C3’H* 5-O-(4-coumaroyl)-D-quinate 3′-monooxygenase, *CHI* chalcone isomerase, *CHS* chalcone synthase, *COMT* caffeate O-methyltransferase, *F3H* flavanone 3-dioxygenase, *FLS* flavonol synthase, *HQT* quinate O-hydroxycinnamoyltransferase, *HCT* shikimate O-hydroxycinnamoyltransferase, *COS* costunolide synthase, *GAO* germacrene A hydroxylase, *GAS* germacrene-A synthase, *GDS* (−)-germacrene D synthase. The colour scale represents the log_2_-transformed TPM value

To investigate the production of valuable secondary metabolites in cardoon, we identified key-genes related transcripts of the phenylpropanoids and flavonoids biosynthetic pathways and analysed their expression patterns during inflorescence development (Fig. 6a, Table S5). We annotated two transcripts for 5-O-(4-coumaroyl)-D-quinate 3′-monooxygenase (C3’H), two for shikimate O-hydroxycinnamoyltransferase (HCT), one for quinate O-hydroxycinnamoyltransferase (HQT), two for caffeate O-methyltransferase (COMT), one for chalcone synthase (CHS) and two for flavonol synthase (FLS). Moreover, the PASA analysis split into two genes the chalcone isomerase (CHI) gene, which was merged in the current annotation. Additionally, the hybrid-seq approach enabled us to detect two new alternatively spliced transcript isoforms for 4-coumarate-CoA ligase the (4CL) gene and one for flavanone 3-dioxygenase (F3H). As regards to pathway regulatory genes, we identified the transcripts homologous to MYB111, MYB308 and MYB86 from *A. thaliana*.

The transcriptome dynamics analysis showed that the genes involved in the early steps of phenylpropanoids and flavonoids biosynthetic pathway, as 4CL, CHS, C3’H and MYB111-like, were up-regulated mostly in first stages of inflorescence development (from CC-1 to CC-3/OC-1) while genes involved in later biosynthesis steps, as F3H and COMT were up-regulated mostly in open capitulum stages (OC-1 to OC-4). Similarly, MYB308-like and MYB86-like showed higher expression levels also at final inflorescence development stages. However, other genes, as CHI, HCT, HQT and FLS showed a wider expression pattern. As for the newly identified isoforms, both for 4CL and F3H, their expression pattern differed across the flower head development stages respect to the isoforms present in the current annotation (Fig. 6a).

### Sesquiterpene lactones biosynthetic pathway

As regards to sesquiterpene lactones biosynthetic pathway, we identified the key-genes related transcripts and analysed their transcriptional dynamics during flower head development (Fig. 6b, Table S5). We annotated two transcripts for germacrene A hydroxylase (GAO), two for (−)-germacrene D synthase (GDS) and two for costunolide synthase (COS). While using the hybrid-seq assembly we detected two alternatively spliced isoforms out of the four annotated transcripts for germacrene-A synthase (GAS). The expression pattern of identified sesquiterpene lactones biosynthetic pathway genes was characterized by an up-regulation mostly in the closed capitulum stages (from CC-1 to CC-3) during inflorescence formation. However, the newly annotated alternatively spliced isoforms showed wider expression pattern respect the transcript already present in the existing annotation.

## Discussion

### Transcriptome analysis through RNA hybrid sequencing

In this study, the joined use of approximately 550 millions reads from SR-seq and 1.4 millions from LR-seq through a hybrid transcriptome assembly approach allowed retrieving full-length transcripts producing 15% more complete assembled genes and 18% more transcript isoforms respect to SR-seq alone. The lower performance of SR-seq alone can be attributed to misassembly of short reads-scaffolds in repetitive regions or problematic reconstruction of lowly expressed transcripts as well as no coverage. The addition of reads from LR-seq, which 90% consisted of full-length transcripts, had a positive impact on improving gene coverage and isoforms detection, providing direct evidence for transcript isoforms of each gene. In total, the assembly quality assessments with rnaQUAST, TransRate and BUSCO tools demonstrated that the hybrid assembly obtained using both LR-seq and SR-seq allowed to retrieve more aligned transcripts and to improve the accuracy and the completeness. In fact, out of 26,889 genes of globe artichoke existing annotation, 13,039 gene models were updated, which mostly accounted for alternative gene structure and UTR modifications at 5′ and 3′ ends of genes. Moreover, 578 new gene models were identified and 11,169 new alternated splicing isoforms were detected, among which intron retention splicing type was the most frequent. Also, functional annotation benefitted from the hybrid assembly with the identification of 1317 more annotated genes and 2249 more GO terms in comparison to the SR-seq assembly. These findings can be both attributed to the genome diversity between globe artichoke and cultivated cardoon and to the more accurate transcriptome annotation obtained with the RNA hybrid sequencing approach.

### Annotation of capitulum development transcriptome

Genes involved in ‘binding’ and ‘catalytic activity’ for molecular function, and ‘metabolic process’ and ‘cellular process’ for biological process were over-represented in inflorescence developing transcriptome. The newly identified gene models exhibited a very similar GO composition as for the unaligned contigs which were analysed as single sequences and not included in the final annotation. The prominence of ‘binding’ term suggested a crucial role of TFs during the flower head development regulation also in Asteraceae as already showed in other plant families [34, 36, 51]. Among the DEGs, approximately two-thirds were TFs including nearly all the transcription factor families. During inflorescence development, TF expression pattern strongly differed between early (closed capitulum samples) and late flower heads maturation stages (open capitulum samples) evidencing the relevant transcriptional programmes changes taking place throughout this process.

Amongst the most represented TFs, bHLH constitute a large family of regulatory proteins found in plants and animals. Some of them were shown to be involved in the regulation of flowering time through the activation of *CONSTANS* gene [52], while others are important in the regulation of flower senescence by modulating ethylene biosynthesis [53]. Here more than half of bHLHs were down-regulated at the CC-1 stage while at OC-2, before anthesis, we observed almost two times more up-regulated genes than down-regulated bHLH genes. This expression pattern, already showed in legumes flowering [36], may suggest the involvement of common bHLHs across plant families during mature flower formation.

MYB transcription factors represent another large TF family playing an important role in flowering development and in secondary metabolites biosynthetic pathways as phenylpropanoids and flavonoids [54, 55]. In the present study, we analysed the differential expression of three MYB TFs likely linked to these pathways in the sampled tissues. MYB111-like, homologous of *AtMYB111*, showed higher expression in the first stages of inflorescence formation, and this may support its key role in the activation of flavonols biosynthesis as documented in artichoke immature inflorescence and young leaves [20]. Conversely, MYB86-like and MYB308-like genes exhibited higher expression levels in opened capitulum stages and this may suggest a role, also for cardoon, as responsible for repression of phenylpropanoids biosynthetic pathway structural genes, i.e. CH4 and 4CL, as previously reported for *A. thaliana* [56, 57].

Moreover, the NAC (NAM, ATAF1,2 and CUC2) and WRKY stress-response related TF families were found to be up-regulated at OC-2 and OC-1 stages, respectively, suggesting their involvement in abiotic (e.g. capitulum wounding and dehydration) and biotic (e.g. insect flower phytophagy) stress response during flowering as described in many plant families [58, 59].

For the MADS family, which is known to play critical roles in orchestrating flowering development, including the floral transition and organogenesis [60], more than half identified TFs were down-regulated at early inflorescence development stages, while they were up-regulated mostly at OC-1, OC-2 stages. This expression pattern already showed in other plant families [61], supports the role of MADS TFs acting down-stream of the floral organ identity factors, also for this species.

### Transcriptional dynamics of identified *C. cardunculus* secondary metabolites

Secondary metabolism of *C. cardunculus* has been of interest in the last decades because of the emerging awareness of polyphenols as healthy compounds [7, 11] and of sesquiterpene lactones as allelochemicals, insect repellents and animal allergens [22, 23]. Expression of polyphenols-related genes was reported to take place during flower development in *Gerbera hybrida* and in *indica* rice [62, 63]. Also, sesquiterpene lactones, consistently with their protecting role, were showed to be produced in artichoke mature leaves and receptacle [28]. For the first time in *C. cardunculus,* in this study the expression dynamics of most of the phenylpropanoids, flavonoids and sesquiterpene lactones biosynthetic pathways related genes were showed through an RNA-seq approach. The biosynthetic genes were strongly expressed during inflorescence development demonstrating the crucial importance of this step in the production of secondary metabolites. Consistently with previous transcriptomic studies in *Lilium* and in *Narcissus* for phenylpropanoids and flavonoids [64, 65] and in *Tanacetum cinerariifolium* for sesquiterpene lactones [66], this approach allowed to functionally annotate these genes in *C. cardunculus* and provides valuable information for further investigations on their role as structural genes in the related pathways. Moreover, the use of RNA hybrid-seq enabled us to identify many alternatively spliced isoforms differentially expressed across the inflorescence development stages showing the complex transcriptional regulation system governing this process. The different expression profiles among isoforms could be associated to different functions carried out throughout the plant life.

## Conclusion

In this study the joined use of reads obtained with LR-seq and SR-seq enhanced transcriptome annotation in terms of accuracy, completeness and isoforms detection for cultivated cardoon, a crop species with a limited genome annotation. To our knowledge, this is the first report showing a hybrid RNA-sequencing approach used for plant inflorescence transcriptome analysis and it is the first transcriptome expression dynamics investigation in this species. This plant, for its ability to grow in dry and marginal lands, has the potential to become a model crop for climate change adaptation, which can be achieved by exploiting genome and transcriptome resources. The proposed approach allowed to substantially improve functional annotation revealing a large number of transcription factors differentially expressed during flower head development. Moreover, the use of hybrid-seq enabled us to detect new alternatively spliced isoforms related to secondary metabolism biosynthetic pathways key genes presenting a differential expression in *C. cardunculus*. A detailed investigation of these pathways exploiting the identified candidate genes and isoforms would be highly desirable to improve the knowledge on the molecular regulation of inflorescence development and of the synthesis of valuable compounds in Cynara.

## Methods

### Plant material and RNA extraction

**Fig. 7:**
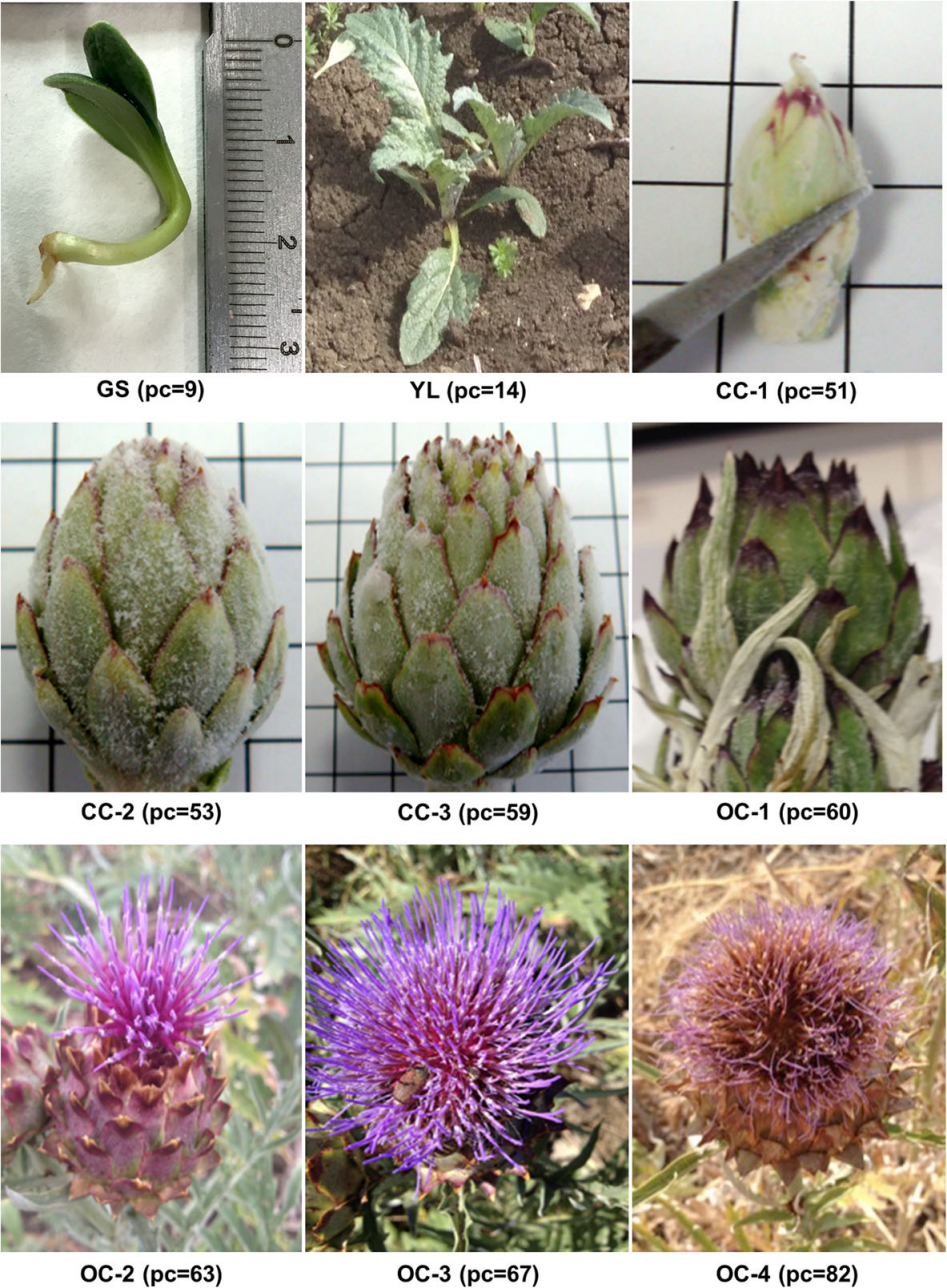
Organ/development stages used for this study with the related phenological code (pc). GS, germinating seedling; YL, young leaves; CC-1 to CC-3, closed capitulum stages; OC-1 to OC-4, open capitulum stages

A transcriptome investigation was carried out taking into consideration different phenological codes (pc) in the growth stages of *Cynara cardunculus* var. *altilis* as reported by Archontoulis et al. [67]. We collected a total of 34 samples with particular attention to inflorescence development including seven stages of floral development, young leaves and seedlings with four biological replicates for each organ/development stage, except for leaves with two replicates each consisting of a pool of two leaves from two different plants (Fig. 7). Only germinating seedlings (GS; pc = 09) were obtained in laboratory imbibing cardoon seeds with water on two #1 Whatman paper filters in Petri dishes at 20 °C with a light/dark 8/16 (hours) photoperiod and collecting seedlings 5 days after germination. The remaining floral and young leaf (YL; pc = 14) samples were collected from field grown plants. The whole inflorescence was collected at seven stages of development including initial closed capitulum (CC-1; pc = 51), visible capitulum (CC-2; pc = 53), enlarged capitulum (CC-3; pc = 59), start of flowering opened capitulum (OC-1; pc = 60), opened capitulum with 30% of heads in blossom (OC-2; pc = 63), opened capitulum in near anthesis with 70% of heads in blossom (OC-3; pc = 67) and opened ripening capitulum with 20–30% of the heads turning yellow (OC-4; pc = 82). For all these samples, the complete collected plant part was immediately immersed and kept in liquid nitrogen until it was ground to a fine powder, of which about 100 mg was used for total RNA isolation. Total RNA was extracted using RNAeasy Plant Mini Kit (Qiagen, Hilden, Germany), with DNase treatment following the manufacturer’s protocol RNA quality and quantity was determined using Eppendorf BioSpectrometer (RNA program) and QIAxcel RNA QC Kit (Qiagen, Hilden, Germany). The extracted total RNA with a RIN/RIS/RQN > 7 was split and processed in-parallel using Illumina and ONT library preparation protocols.

### Short read sequencing

Library preparation and sequencing were outsourced (Eurofins GmbH, Ebersberg, Germany). For each sample, approximately 1 μg of total RNA were used for library preparation applying a strand-specific cDNA libraries synthesis kit (New England Biolabs, Ipswich, USA). The mRNA was selected with a polyA capturing method, fragmented, ligated with adapters and amplified [68]. Samples from each library were pooled equimolar and paired-end (PE) sequenced using HiSeq2500 (Illumina Technologies, San Diego, USA) platform with chemistry v4 applying the high-output run mode. The obtained reads were used as short read-sequencing (SR-seq) reads for downstream analyses.

### Long read sequencing

We modified and adapted the ONT Nanopore cDNA-PCR Sequencing Kit SQK-PCS108 (Oxford Nanopore Technologies, Oxford, UK) for the preparation of library starting from total RNA as reported in Picelli et al. [69, 70]. To ensure a representative sequencing of transcript isoforms, many of which may be tissue-specific, we multiplexed all sampled tissues and pooled them for subsequent sequencing. In brief, 600 ng of total RNA was denatured at 65 °C for 5 min for Oligo-dT30VN hybridization, then reverse transcribed using the strand-switching protocol in 20 μl of the reaction mixture as reported in the relevant cDNA-PCR sequencing kit protocol (version: PCS_9035_v108_revF, Oxford Nanopore Technologies, Oxford, UK). The reaction was incubated at 42 °C for 40 min then with 10 cycles of (50 °C for 2 min, 42 °C for 2 min) to optimize template-switching followed by 1 cycle of (80 °C for 10 min) for enzyme inactivation. The double-stranded cDNA was split into four PCR reactions for which we used Long AmpTaq 2x MasterMix (NEB) and cDNA primer (cPrim) for amplification incubating at 95 °C for 30 s followed by 20 cycles of (95 °C for 15 s, 50 °C- 55 °C for 15 s, 65 °C for 3 min), with a final extension at 65 °C for 6 min. Purification steps were carried out following manufacturer’s instructions and the resulting cDNA was evaluated and quantified using an Agilent High Sensitivity DNA kit and Chip on a Bioanalyzer 2100 (Agilent Technologies, Santa Clara, CA, USA). Adapter (cAMX) was added to 23 μl of amplified cDNA library then the reaction was purified with 1x volume Agencourt AMPure XP beads (Beckman Coulter, Beverly, USA), according to ONT protocol. The purified library was quantified by fluorometric quantitation and 600 fmol was mixed with library loading beads and running buffer with fuel mix provided in ONT protocol. The full-length cDNA library was then sequenced on a MinION R9.5 flow cells for 48 h using the 1D sequencing protocol. The obtained reads were used as long-sequencing reads (LR-seq) for downstream analyses.

### Data analysis

#### Quality control

Both the results of SR-seq and LR-seq were mapped to v.2 of *C. cardunculus* genome available at (www.artichokegenome.unito.it) with STAR [71] and minimap2 [72] in spliced alignment mode respectively to assess their quality.

### SR-seq reads analysis

Illumina paired-end 150 bp reads in FASTQ format were analysed with the FastQC program [73], and then quality and adaptors, barcodes, polyA and polyT ends were trimmed using Cutadapt v1.16 [74] with default parameters for paired-end reads and Trimmomatic v0.33 [75] in paired-end mode, setting the minimum length to 50 bp. De novo assembly was performed using rnaSPAdes from SPAdes 3.12 package [76] with default parameters.

### Hybrid-seq assembly

To enable the use of LR-seq reads to obtain a hybrid-seq transcriptome assembly we combined the existing rnaSPAdes [49] and hybridSPAdes [77] approaches, with the former developed for assembling short-read RNA-Seq data and the latter one designed for hybrid genome assembly. The complete algorithm description can be found in the separate software publication [78]. To obtain high-quality transcriptome assembly in the present study we customized several important settings combining short and long reads (Figure S1). We took advantage of full-length (FL) transcripts coverage of LR-seq by mapping the ONT reads to the SR-seq assembly using BWA-MEM [79] without setting any minimum length threshold on the resulting assembly graph since for transcriptomic data it is typically more fragmented. To improve isoforms detection, we extended the reads path modifying the exSPAnder module [50] in order to process multiple edges simultaneously. This allowed to extend each path individually and to detect separate alternative isoforms. In addition, LR-seq reads were analysed to detect sequences corresponding to full-length transcripts and the paths obtained from FL reads were also marked as full-length for use in further analyses. The read was considered as FL when both adapters were detected based on sequence alignment with at least 70% similarity. FL reads were processed in the same way as raw LR-seq reads, but the paths obtained from FL read mapping were also added directly to the resulting set of paths. To avoid excessive transcript sequences in the resulting FASTA file, we also removed duplicated paths before outputting them.

### Quality assessment of de novo assembly

De novo assembly of sequencing data was performed in order to obtain aberrant and new genes and isoforms that are missing from the existing gene database available for this species. The quality evaluation of obtained assemblies (from SR-seq and hybrid-seq) was performed using several different approaches. We used the published V2 artichoke genome ([29, 30]; www.artichokegenome.unito.it, downloaded in October 2018) and its gene annotation to assess quality with respect to known genes rnaQUAST v1.5.1 [80]. The transcript dataset was assessed with Benchmarking Universal Single-Copy Orthologs, BUSCO [81], to estimate the number of plant universal single-copy orthologs (core genes) in the assemblies and TransRate v1.0.3 [82] was run to evaluate the completeness and accuracy of the assembled transcripts measuring the quality of individual contig and of the whole assembly.

### Transcriptome annotation

The obtained assemblies, hybrid-seq and SR-seq, were aligned to the reference genome and refined into complete gene models including alternatively spliced isoforms using PASA pipeline v.2.0.0 (Program to Assemble Spliced Alignments, [83]). Transcripts that failed to align to the reference genome sequence were removed from the dataset and in silico functionally characterized to find out possible novel annotations. Using the aligned transcripts, PASA generates an updated annotation by extending and refining the existing gene database with new gene models, UTRs, newly detected alternatively spliced isoforms and novel genes. To analyse the effect of the two sequencing approaches on functional annotation, we aligned the SR-seq and hybrid-seq transcriptomes, refined by PASA, to the publicly available protein databases including NCBI non-redundant (nr) protein database (downloaded in December 2018), using a local BLASTX analysis with an *E* value cut-off of 10^− 5^ and using InterProScan to infer protein function. The results were used with Blast2Go suite program [84] using default parameters to retrieve GO terms and enzyme codes and to visualize specific pathways loaded from Kyoto Encyclopedia of Genes and Genomes (KEGG). Then, we used the WEGO web application [85] to carry out a comparative analysis between the SR-seq and hybrid-seq GO annotations showing gene numbers and percentages of differing GO terms setting up a *p* value ≤0.05.

The composition of genes during the plant growth stages was investigated through an enrichment analysis of DEG using the Fisher’s Exact test and False Discovery Rate (FDR) considering the list of DEGs as “test-set” and the annotated transcriptome obtained with the hybrid approach as “reference-set”. The enriched GO list was, then, analysed with the AgriGO web application [86], with Benjamini-Hochberg correction (*p* value ≤0.01) to limit the representation to the most enriched terms.

### Differential expression analysis

To quantify cultivated cardoon transcript expressions, we aligned pre-processed quality-trimmed reads on the reference genome and we calculated the expression values with the aligned read counts for each transcript. HiSat2 software [87] was used to align the reads on the transcript sequences and HtSeq count [88] was used to evaluate gene expression, in terms of Transcripts per Millions (TPM), from the aligned results. The analysis of differentially expressed genes (DEGs) was carried out with edgeR R package [89] following manual directions for testing multiple conditions. The general DEGs list was obtained comparing all the samples together and this list was used to perform the enrichment GO analysis as “test set” compared to the updated annotation used as “reference set”. To analyse the flowering-related DEGs we compared each inflorescence developmental stage with the previous one. In each analysis, a criterion of |log_2_(Ratio)| ≥ 2 and an FDR of ≤0.01 was used. The resulting DEGs set was used to identify most differentially expressed transcription factors (TFs) by searching for sequence homologs in the v4.0 Plant Transcription Factor Database (www.planttfdb.cbi.pku.edu.cn; downloaded in January 2019) [90] with local BLASTX (*E* value cut-off of 10^− 5^). To determine expression patterns of selected genes in the phenylpropanoid, flavonoid and sesquiterpene lactones biosynthetic pathways we identified matching transcripts and their expression levels were plotted as heat maps across phenological stages using log_2_ normalized counts.

### Quantitative PCR analysis

To validate the expression profiles of RNA-seq data, we prepared cDNA from 500 ng of total RNA of each sampled tissue using the QuantiTect® Kit (Qiagen, Hilden, Germany) following manufacturer’s instructions. Then, we performed real-time PCR reactions with the QuantiNova SYBR Green® Kit (Qiagen, Hilden, Germany): in brief, for each reaction we used 1 μl of cDNA, forward and reverse primer at final concentration 0.7 μM each and 10 μl Sybr Green RT-PCR Master Mix in a total volume of 20 μl. RT-qPCRs were carried out on a Rotorgene 6000 cycler (Qiagen, Hilden, Germany) with the following cycling parameters: initial denaturation of 2′ at 95 °C then 35 amplification cycles of 2″ at 95 °C and 10″ at 60 °C. Quantitative analysis were performed on seven genes selected among the identified phenylpropanoid, flavonoid and sesquiterpene lactones biosynthetic pathways DEGs using three independent biological replicates and three technical replicates of each biological replicates for each tissue sample. The primer sequences used for real-time PCR analysis in this study were designed using Primer 3 [91] and are given in Table S1. The actin gene was used as housekeeping gene and the fold change in all tissues for each gene was calculated with respect to GS sample. All RT-qPCR data were submitted to the Bartlett’s test for the homogeneity of variance and then analysed using one way analysis of variance (ANOVA), (*p* value ≤0.05), with the CoStat software (CoHort software, Monterey, CA, USA). The correlation between expression profiles of the selected genes measured by qRT-PCR and RNA-seq was calculated with R software.

## Supplementary information

**Additional file 1: Figure S1.** Hybrid RNA-seq assembly pipeline. (a) The reads from Illumina SR-seq are used to obtain de novo contigs from short reads (via De Bruijn graph) reconstructing full-length transcript (1) with potential alternatively spliced isoform remaining unassembled (2a). (b) the reads obtained from LR-seq are aligned to contigs from short reads reconstructing complete genes retrieving full-length alternatively spliced isoforms (‘1’ and ‘2b’).

**Additional file 2: Figure S2.** Gene Ontology (GO) analysis of the assemblies obtained using hybrid-seq (A) and with SR-seq only (B).

**Additional file 3: Figure S3.** Gene Ontology (GO) analysis of the 578 new gene models identified.

**Additional file 4: Figure S4.** Hit species distribution of the unaligned contigs obtained with BLASTX analysis.

**Additional file 5: Figure S5.** Gene Ontology (GO) analysis of the unaligned contigs.

**Additional file 6: Figure S6.** Principal component analysis showing the cluster separation among the samples.

**Additional file 7: Figure S7.** Quantitative RT-PCR validation of differential gene expression. Relative transcript abundance of 7 differential expressed genes validated by real-time PCR analysis is shown. The fold change in all tissues/stages for each gene was calculated with respect to GS sample. GS, germinating seedling; YL, young leaf; CC1-CC3 closed capitulum stages; OC1-OC4 open inflorescence stages. The error bars represents mean ± standard deviation. Letters indicate only significantly different values according to ANOVA (*p* value ≤0.05).

**Additional file 8: Figure S8.** Correlation of gene expression results obtained from real-time PCR analysis and RNA-seq (TPM) for 7 selected genes in 9 tissue samples. The correlation of determination (R^2^) was 0.72.

**Additional file 9: Figure S9.** Hierarchical cluster analysis of differentially expressed genes across all the phenological stages (GS, YL, CC1, CC2, CC3, OC1, OC2, OC3 and OC4).

**Additional file 10: Figure S10.** GO enrichment analysis of 2968 DEGs with the updated annotation used as reference.

**Additional file 11: Figure S11.** Acyclic graphs relative to enrichment analysis of hybrid assembly for biological processes (a), molecular functions (b) and cellular components (c).

**Additional file 12: Figure S12.** Hierarchical cluster analysis of differentially expressed genes across inflorescence development stages (CC1, CC2, CC3, OC1, OC2, OC3 and OC4).

**Additional file 13: Figure S13.** Expression levels of all the identified transcription families across the inflorescence development stages.

**Additional file 14: Figure S14.** Number of genes for each differentially expressed transcription factor family across inflorescence development.

**Additional file 15: Table S1.** Primer sequences used for validation of RNA-seq data using qRT-PCR reactions.

**Additional file 16: Table S2**. RNA-seq data obtained with Illumina (SR-seq) and ONT (LR-seq) platforms.

**Additional file 17: Table S3.** Comparison of annotations of SR-seq and Hybrid-seq assemblies refined and updated by PASA software.

**Additional file 18: Table S4.** Functional annotation comparison of the transcriptome obtained using hybrid seq and SR-seq.

**Additional file 19: Table S5.** BLASTX result of phenylpropanoids, flavonoids and sesquiterpene lactones key genes detected in this study.

## Data Availability

The datasets generated and analysed in the current study are available in the NCBI SRA repository PRJNA590905 (https://www.ncbi.nlm.nih.gov/bioproject/PRJNA590905).

## Abbreviations

4CL: 4-coumarate-CoA
bHLH: Basic helix-loop-helix
BLAST: Basic Local Alignment Search Tool
C3’H: 5-O-(4-coumaroyl)-D-quinate 3′-monooxygenase
CC: Closed capitulum
CHI: Chalcone isomerase
CHS: Chalcone synthase
COMT: Caffeate O-methyltransferase
COS: Costunolide synthase
CQA: Caffeoylquinic acids
DEG: Differentially expressed genes
F3’H: flavonoid 3′-hydroxylase
F3H: Flavanone 3-dioxygenase
FDR: False discovery rate
FL transcripts: Full-length transcripts
FLS: Flavonol synthase
GAO: Germacrene A hydroxylase
GAS: Germacrene synthase A
GDS: (−)-Germacrene D synthase
GO: Gene Ontology
GS: Germinating seedlings
HCT: Shikimate O-hydroxycinnamoyltransferase
HQT: Quinate O-hydroxycinnamoyltransferase
Hybrid-seq assembly: Hybrid assembly using both short and long reads
KEGG: Kyoto Encyclopedia of Genes and Genomes
LR-seq: Short read sequencing using ONT Nanopore platform
OC: Open capitulum
PASA: Program to Assemble Spliced Alignments
PC: Phenological codes
PE: Paired-end
ROS: Reactive oxygen species
SR-seq: Short-read sequencing using Illumina platform
TF: Transcription factor
TPM: Transcripts per millions
UTR: Untranslated region
YL: Young leaves
