# Supplementary figures and images for: Hybrid transcriptome sequencing approach improved assembly and gene annotation in Cynara cardunculus (L.)
Source: BMC Genomics. 2020 Aug 21;21:317. doi: 10.1186/s12864-020-6670-5 (PMC7441626; doi:10.1186/s12864-020-6670-5)

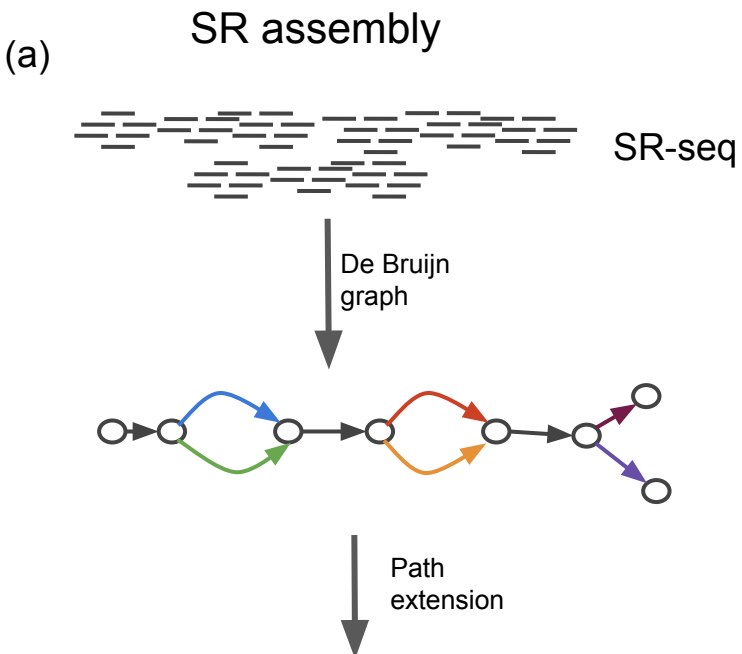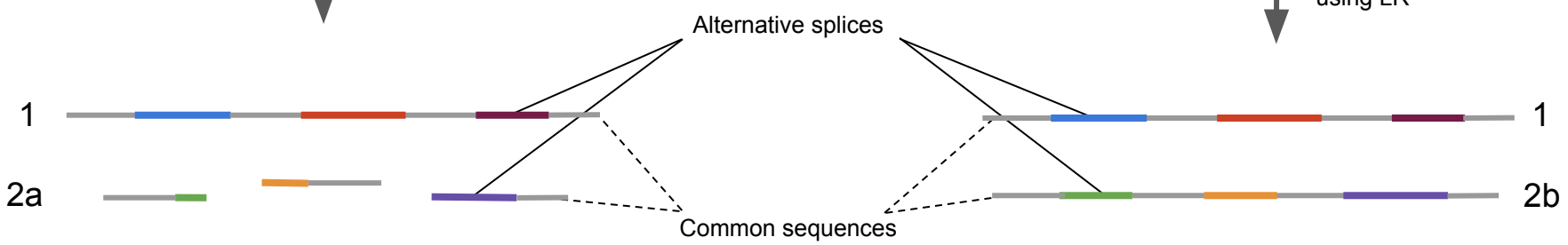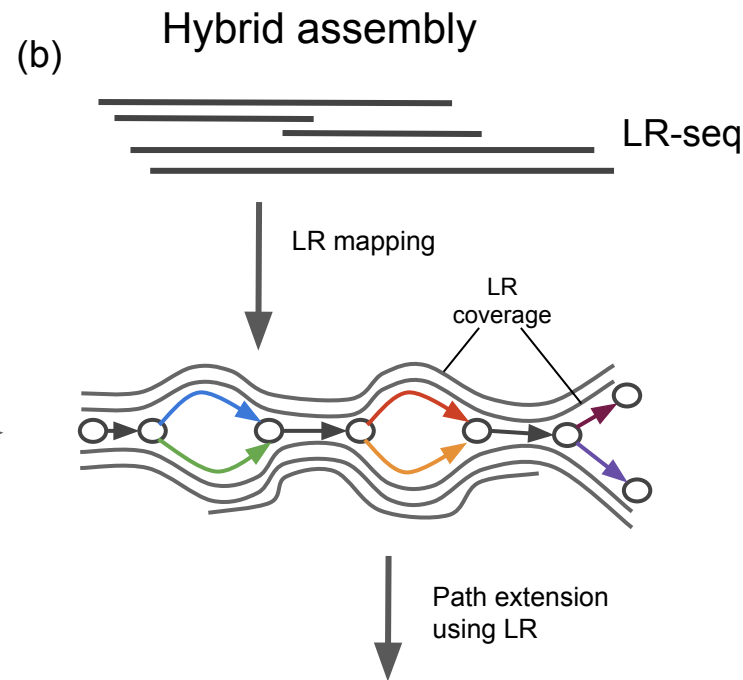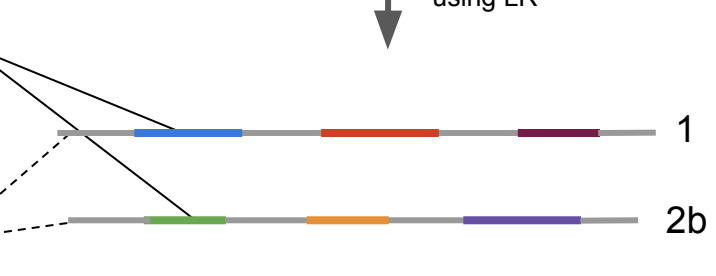

Supplement: Supplementary file 1 — Additional file 1: Figure S1. Hybrid RNA-seq assembly pipeline. (a) The reads from Illumina SR-seq are used to obtain de novo contigs from short reads (via De Bruijn graph) reconstructing full-length transcript (1) with potential alternatively spliced isoform remaining unassembled (2a). (b) the reads obtained from LR-seq are aligned to contigs from short reads reconstructing complete genes retrieving full-length alternatively spliced isoforms (‘1’ and ‘2b’). [file 12864_2020_6670_MOESM1_ESM.pdf]

A

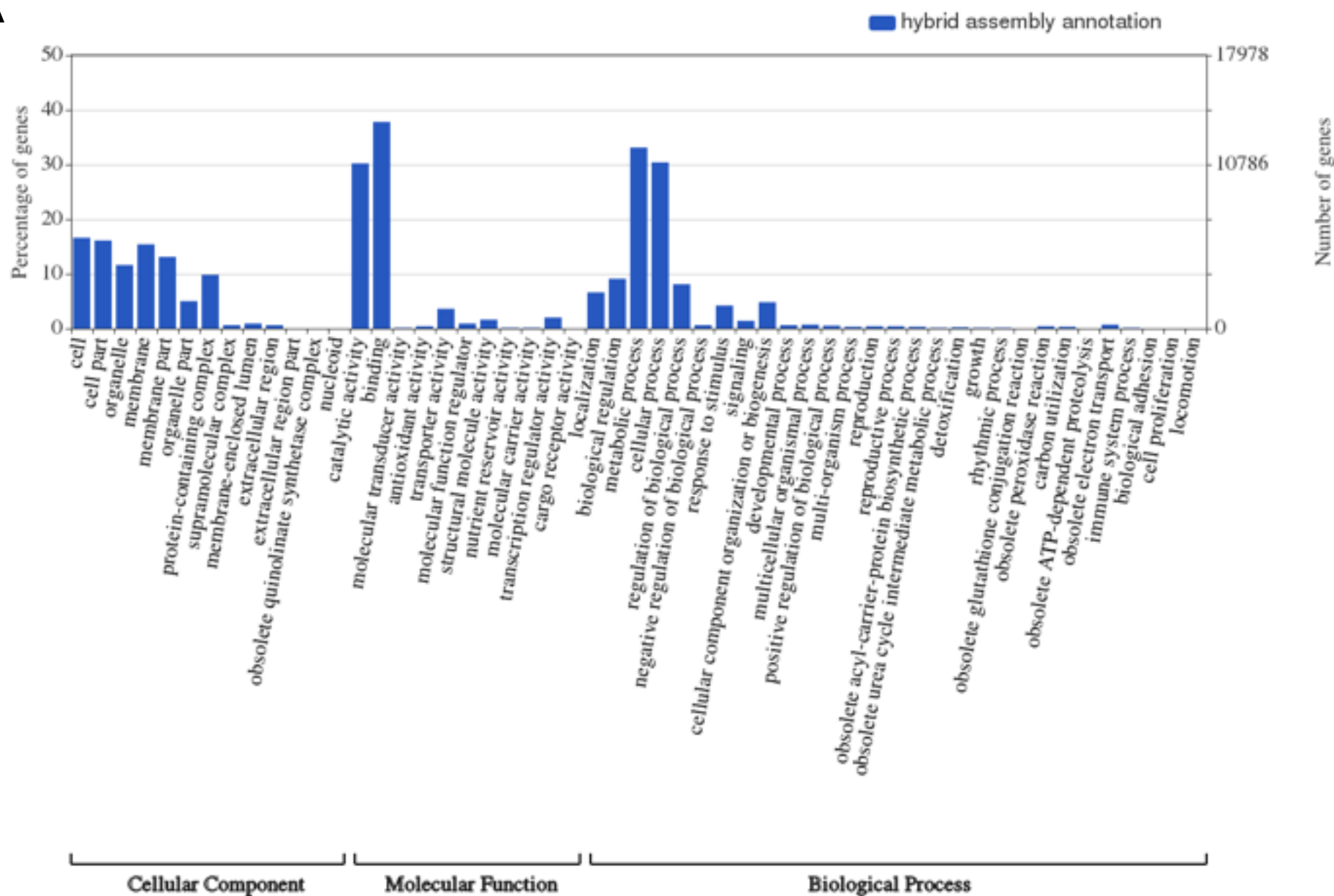

B

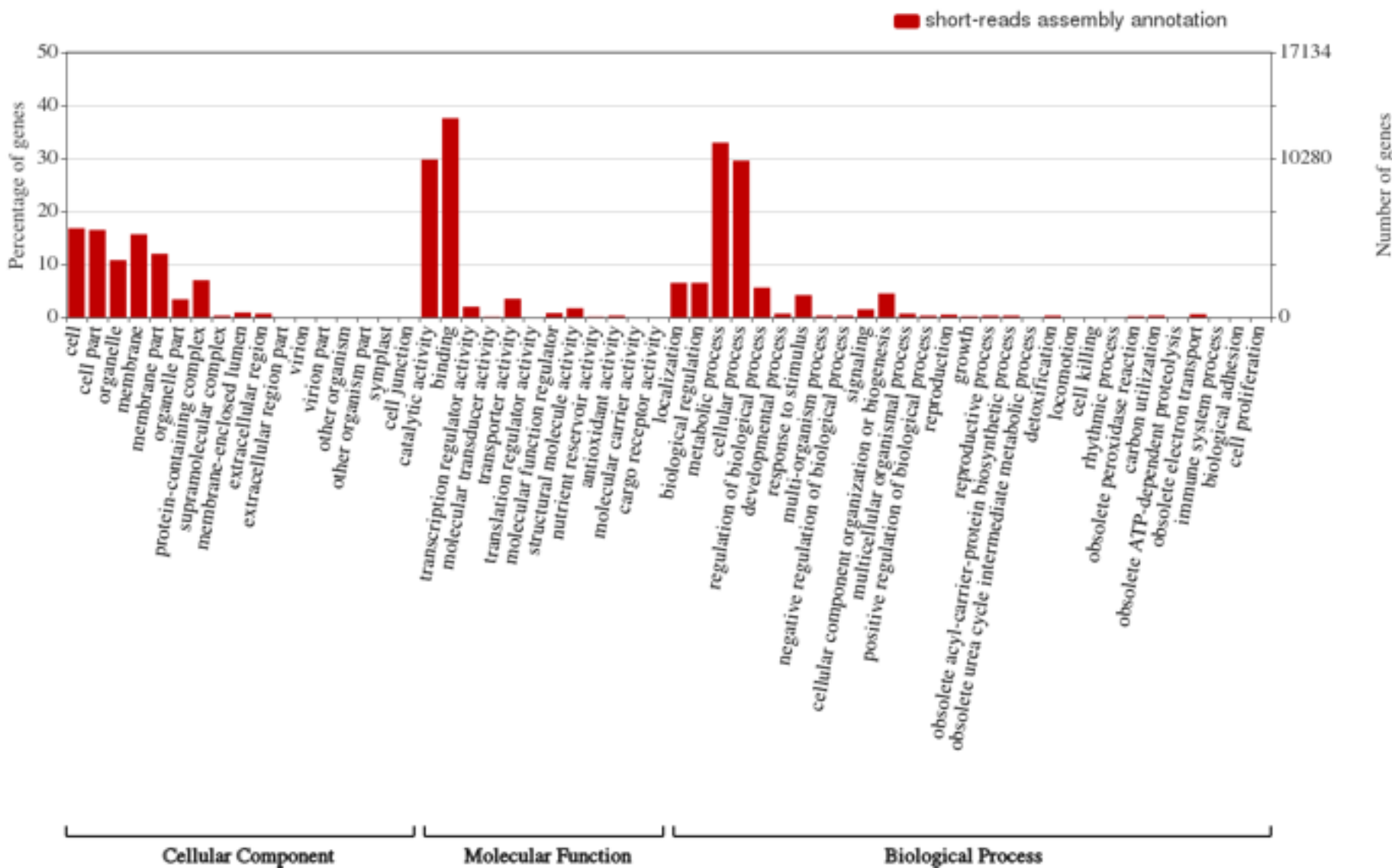

Supplement: Supplementary file 2 — Additional file 2: Figure S2. Gene Ontology (GO) analysis of the assemblies obtained using hybrid-seq (A) and with SR-seq only (B). [file 12864_2020_6670_MOESM2_ESM.pdf]

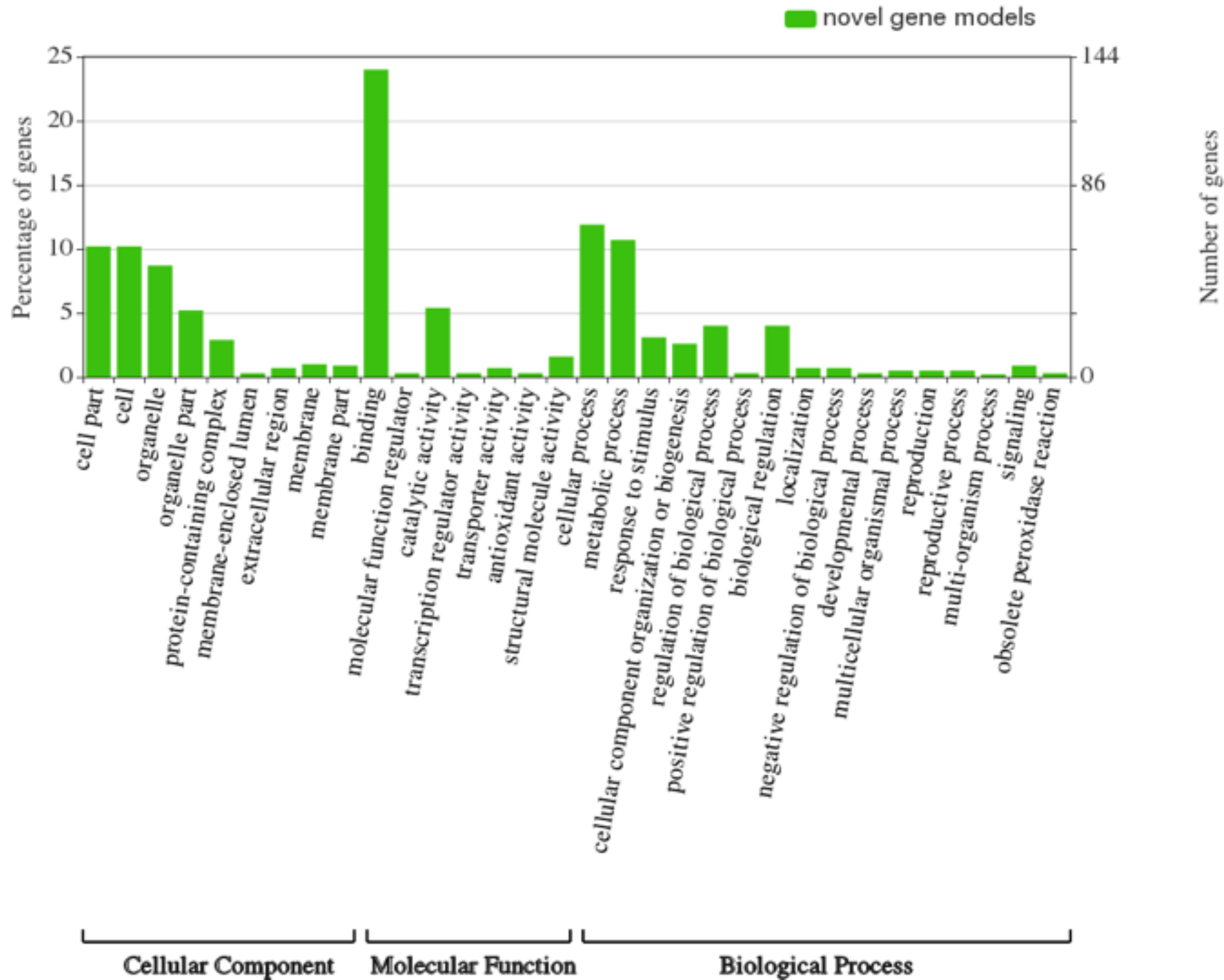

Supplement: Supplementary file 3 — Additional file 3: Figure S3. Gene Ontology (GO) analysis of the 578 new gene models identified. [file 12864_2020_6670_MOESM3_ESM.pdf]

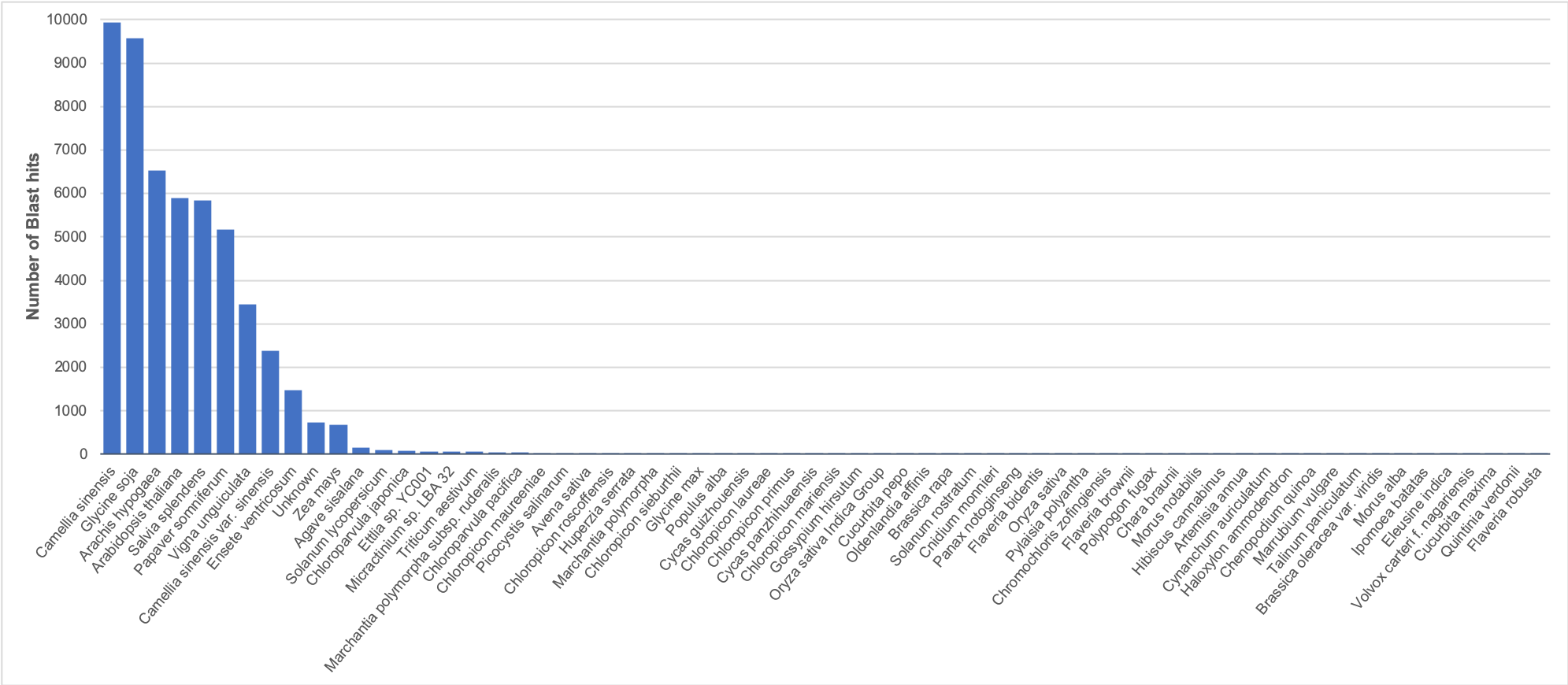

Supplement: Supplementary file 4 — Additional file 4: Figure S4. Hit species distribution of the unaligned contigs obtained with BLASTX analysis. [file 12864_2020_6670_MOESM4_ESM.png]

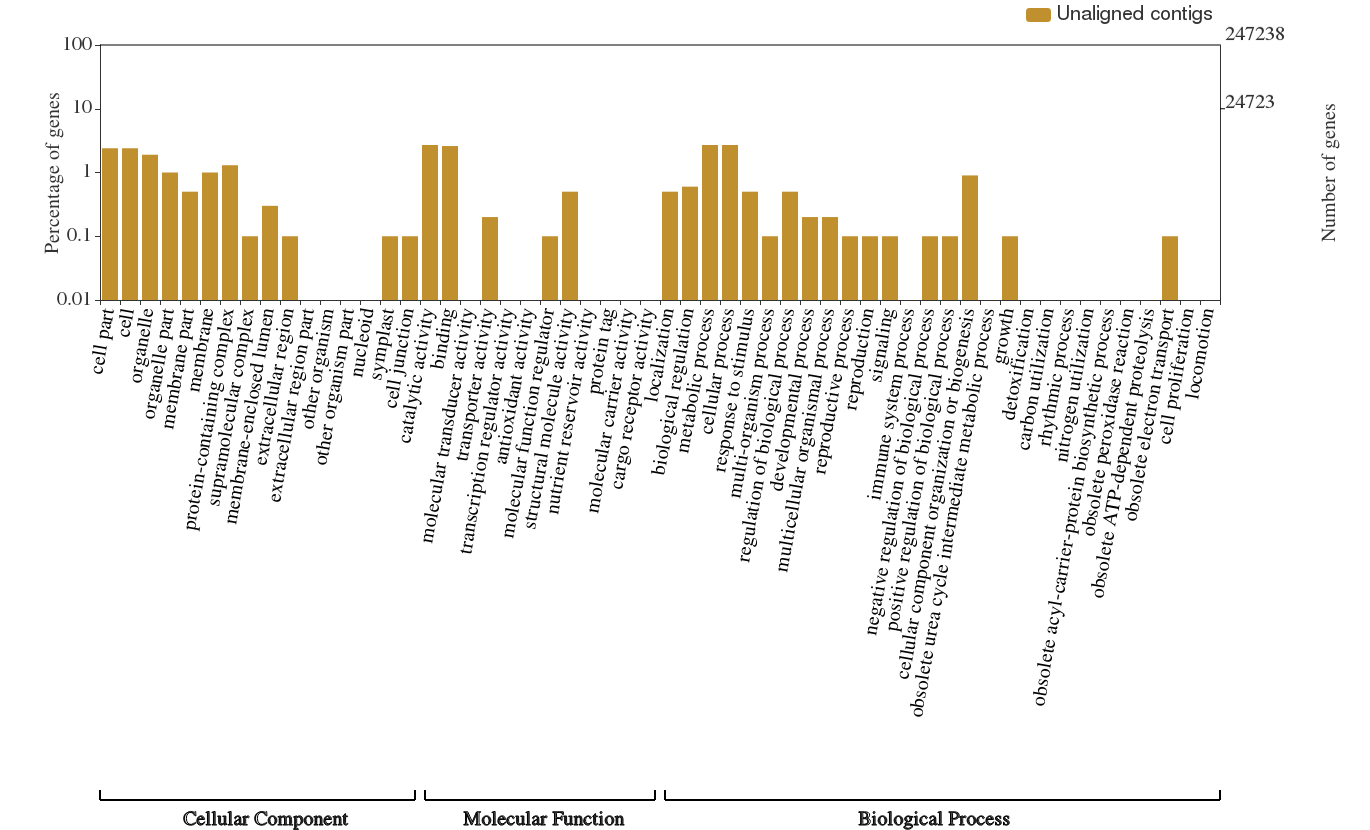

Supplement: Supplementary file 5 — Additional file 5: Figure S5. Gene Ontology (GO) analysis of the unaligned contigs. [file 12864_2020_6670_MOESM5_ESM.png]

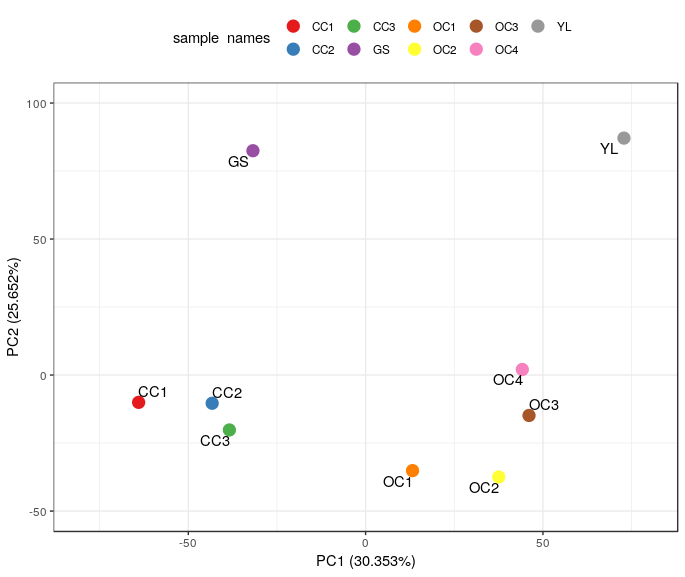

Supplement: Supplementary file 6 — Additional file 6: Figure S6. Principal component analysis showing the cluster separation among the samples. [file 12864_2020_6670_MOESM6_ESM.png]

Relative transcript abundance (fold change)

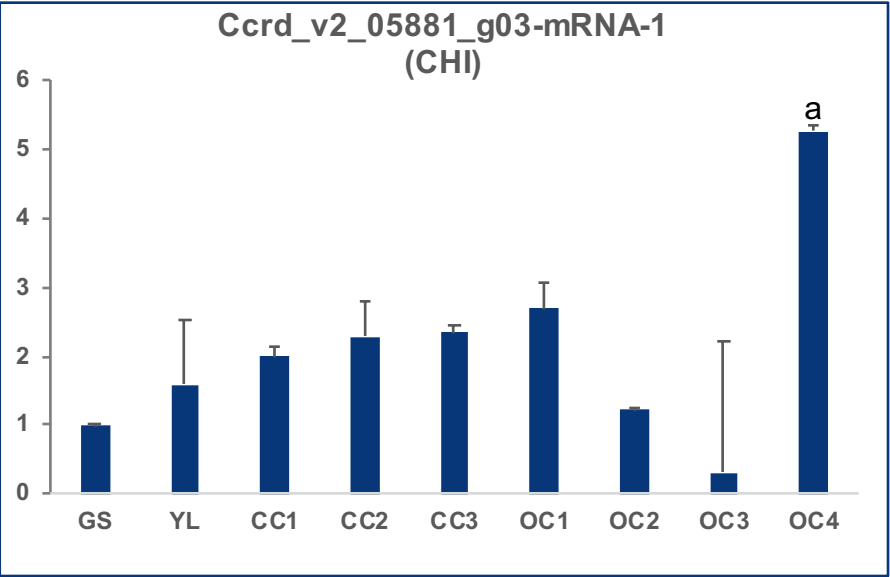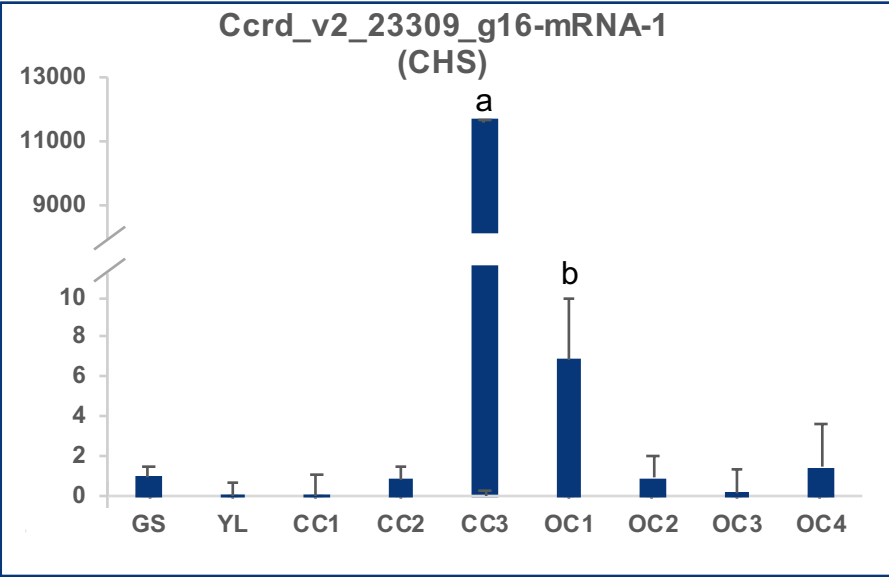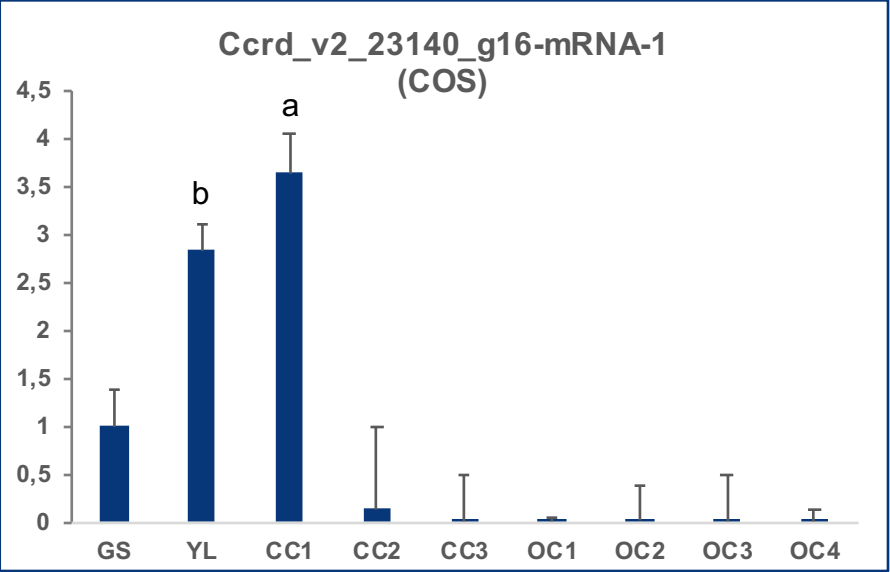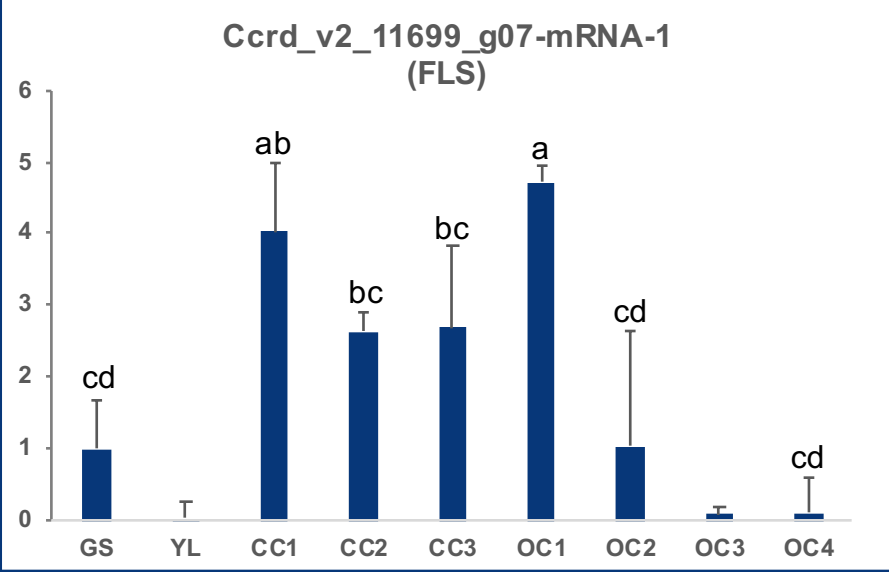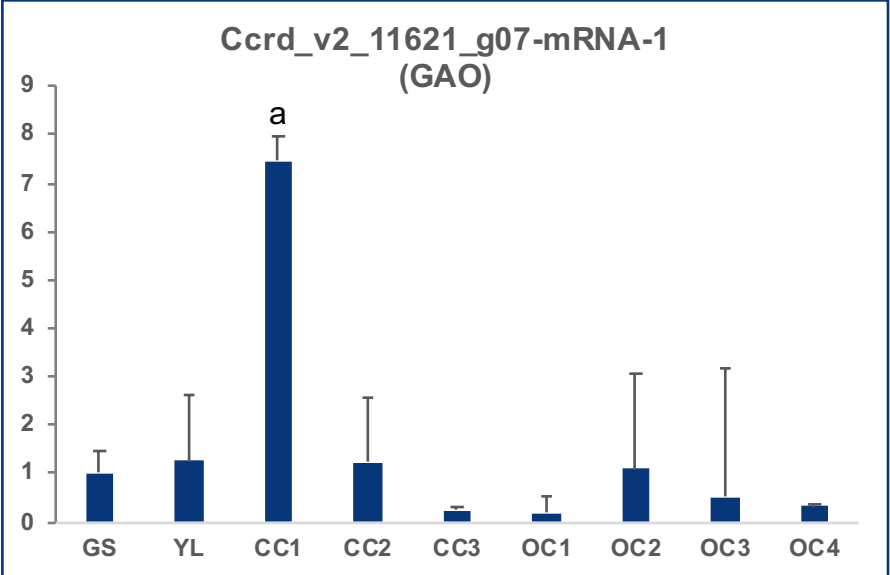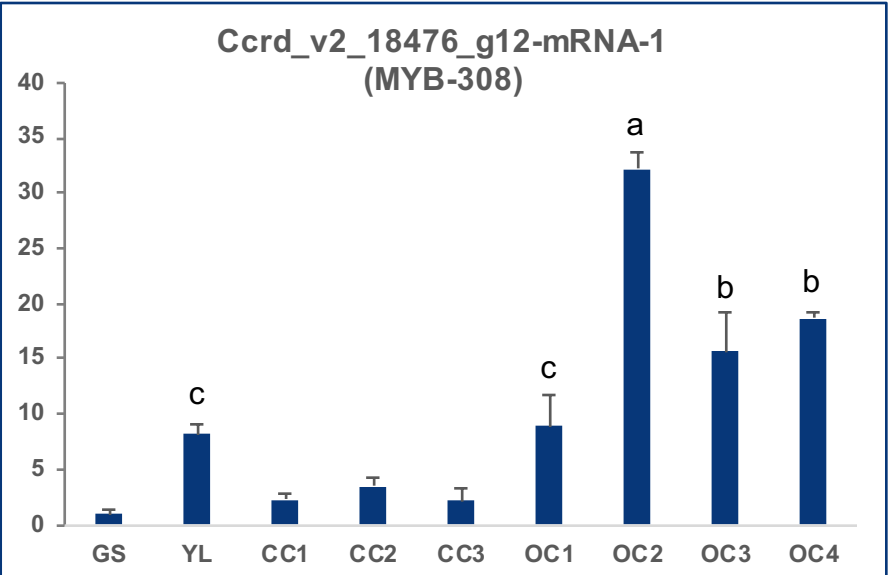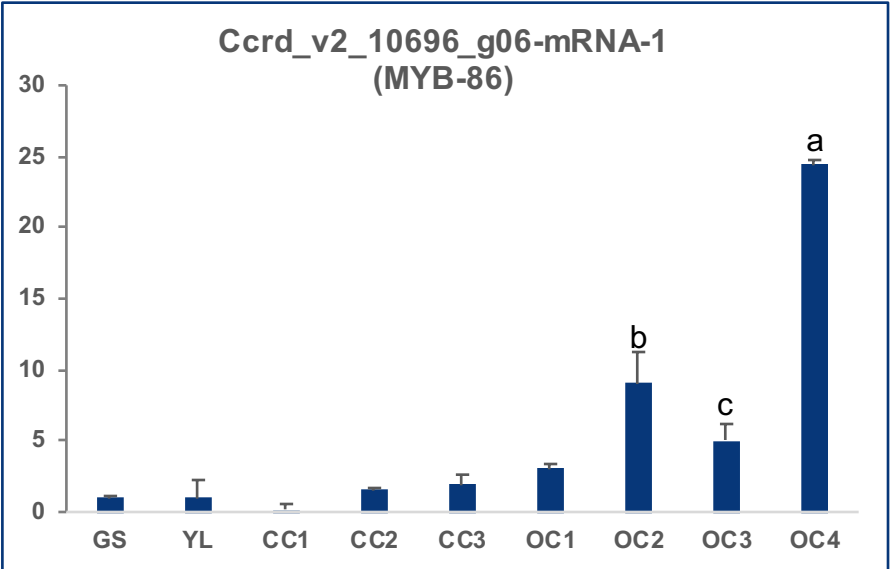

Supplement: Supplementary file 7 — Additional file 7: Figure S7. Quantitative RT-PCR validation of differential gene expression. Relative transcript abundance of 7 differential expressed genes validated by real-time PCR analysis is shown. The fold change in all tissues/stages for each gene was calculated with respect to GS sample. GS, germinating seedling; YL, young leaf; CC1-CC3 closed capitulum stages; OC1-OC4 open inflorescence stages. The error bars represents mean ± standard deviation. Letters indicate only significantly different values according to ANOVA (p value ≤0.05). [file 12864_2020_6670_MOESM7_ESM.pdf]

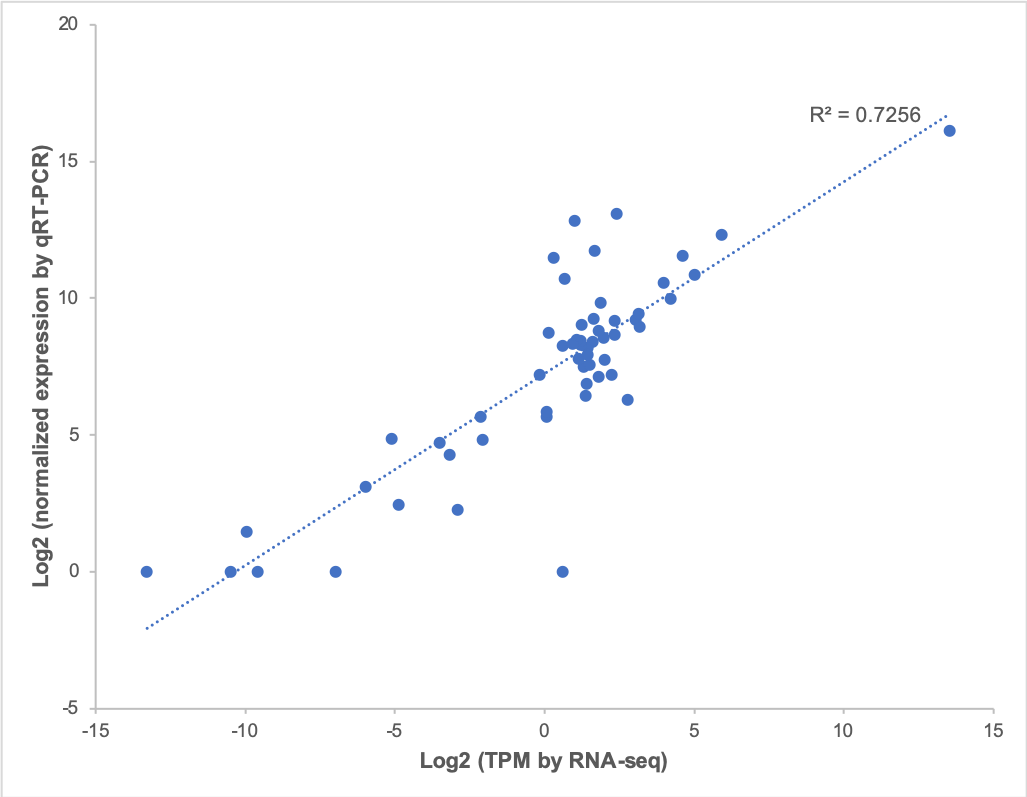

Supplement: Supplementary file 8 — Additional file 8: Figure S8. Correlation of gene expression results obtained from real-time PCR analysis and RNA-seq (TPM) for 7 selected genes in 9 tissue samples. The correlation of determination (R2) was 0.72. [file 12864_2020_6670_MOESM8_ESM.png]

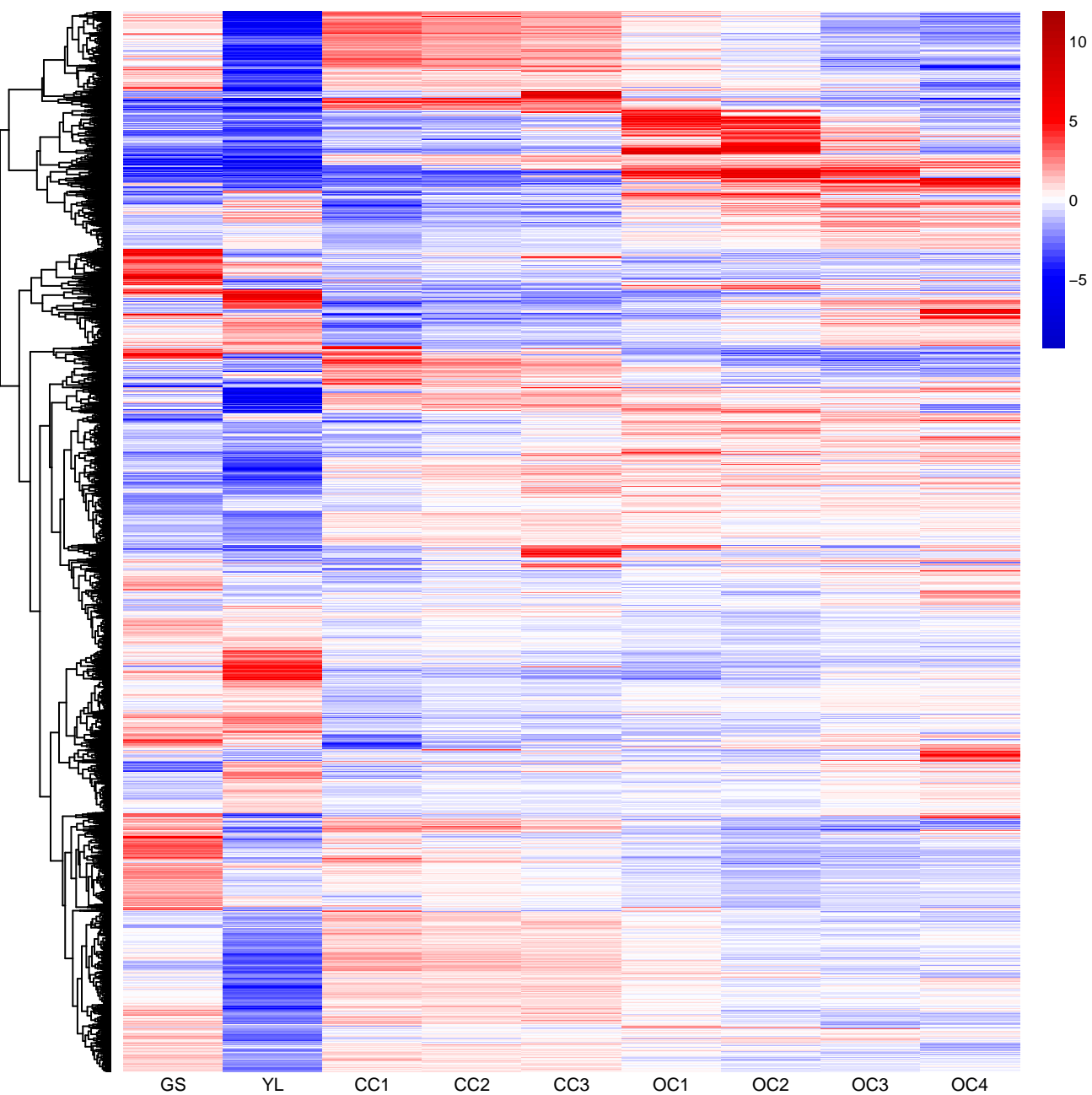

Supplement: Supplementary file 9 — Additional file 9: Figure S9. Hierarchical cluster analysis of differentially expressed genes across all the phenological stages (GS, YL, CC1, CC2, CC3, OC1, OC2, OC3 and OC4). [file 12864_2020_6670_MOESM9_ESM.pdf]

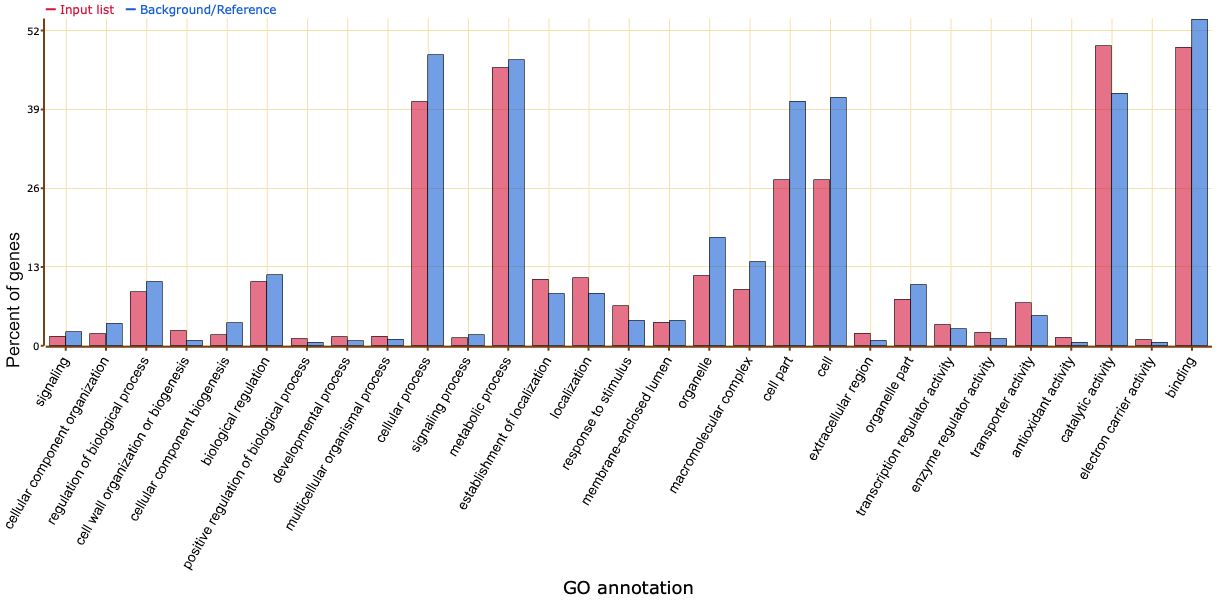

Supplement: Supplementary file 10 — Additional file 10: Figure S10. GO enrichment analysis of 2968 DEGs with the updated annotation used as reference. [file 12864_2020_6670_MOESM10_ESM.png]

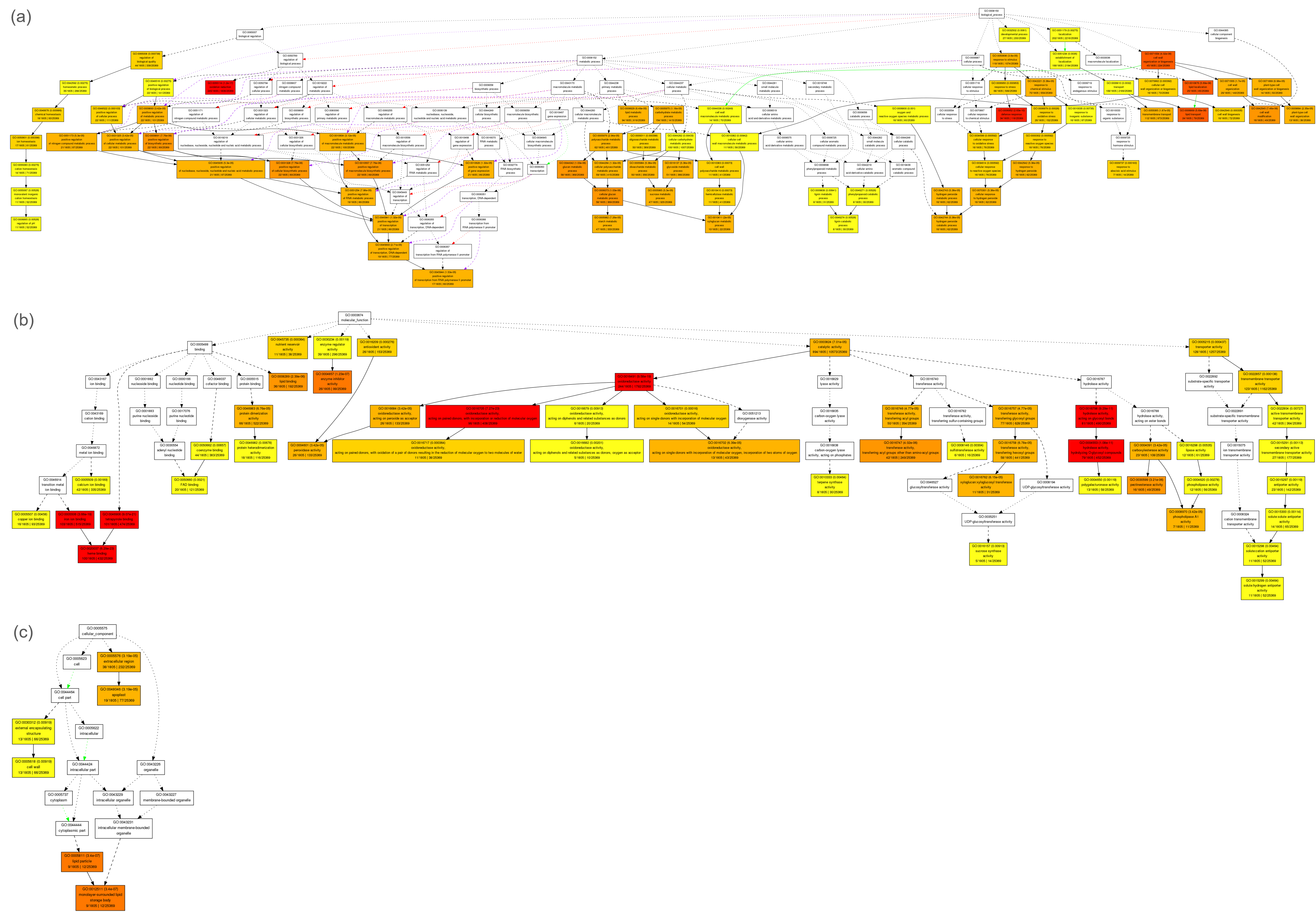

Supplement: Supplementary file 11 — Additional file 11: Figure S11. Acyclic graphs relative to enrichment analysis of hybrid assembly for biological processes (a), molecular functions (b) and cellular components (c). [file 12864_2020_6670_MOESM11_ESM.tif]

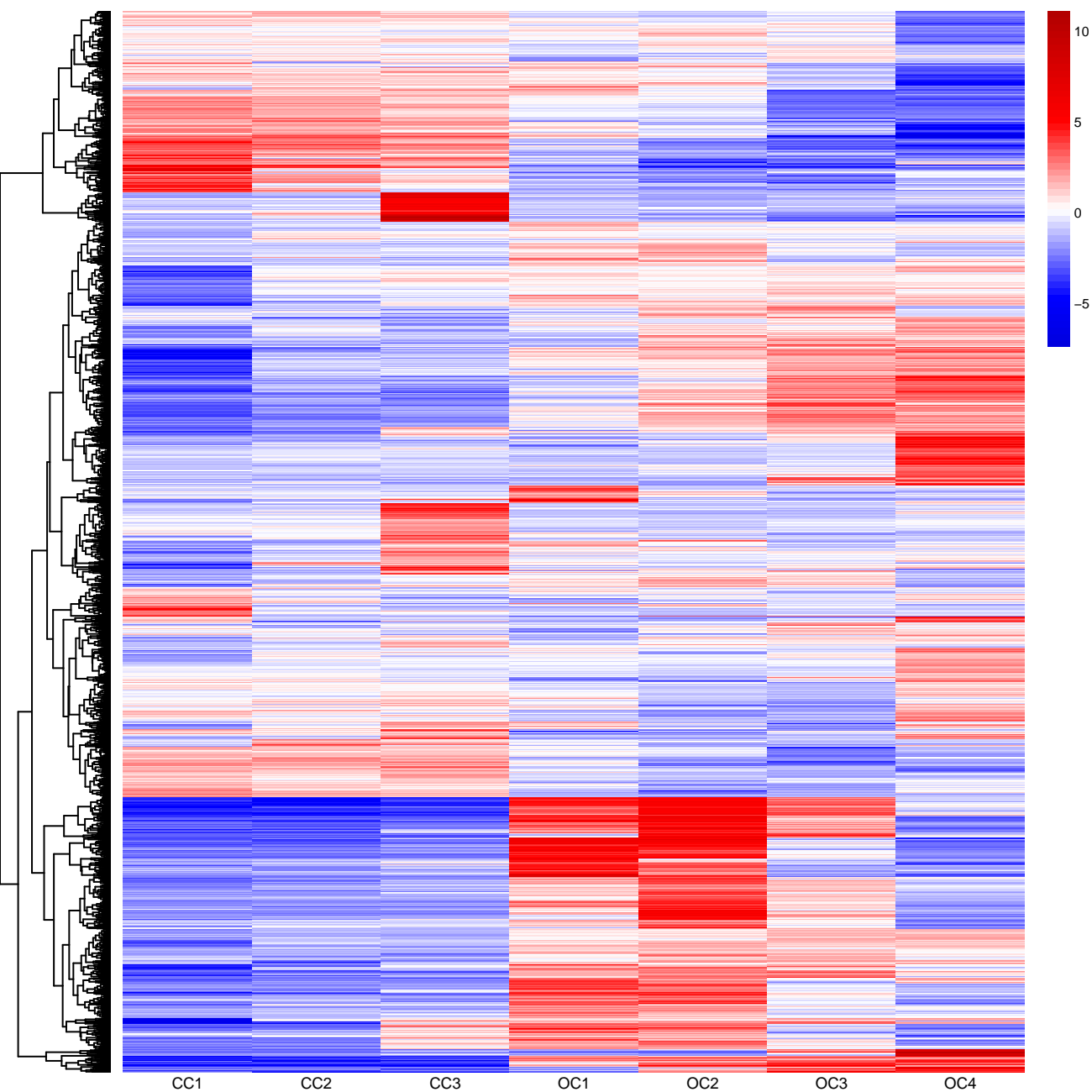

Supplement: Supplementary file 12 — Additional file 12: Figure S12. Hierarchical cluster analysis of differentially expressed genes across inflorescence development stages (CC1, CC2, CC3, OC1, OC2, OC3 and OC4). [file 12864_2020_6670_MOESM12_ESM.pdf]

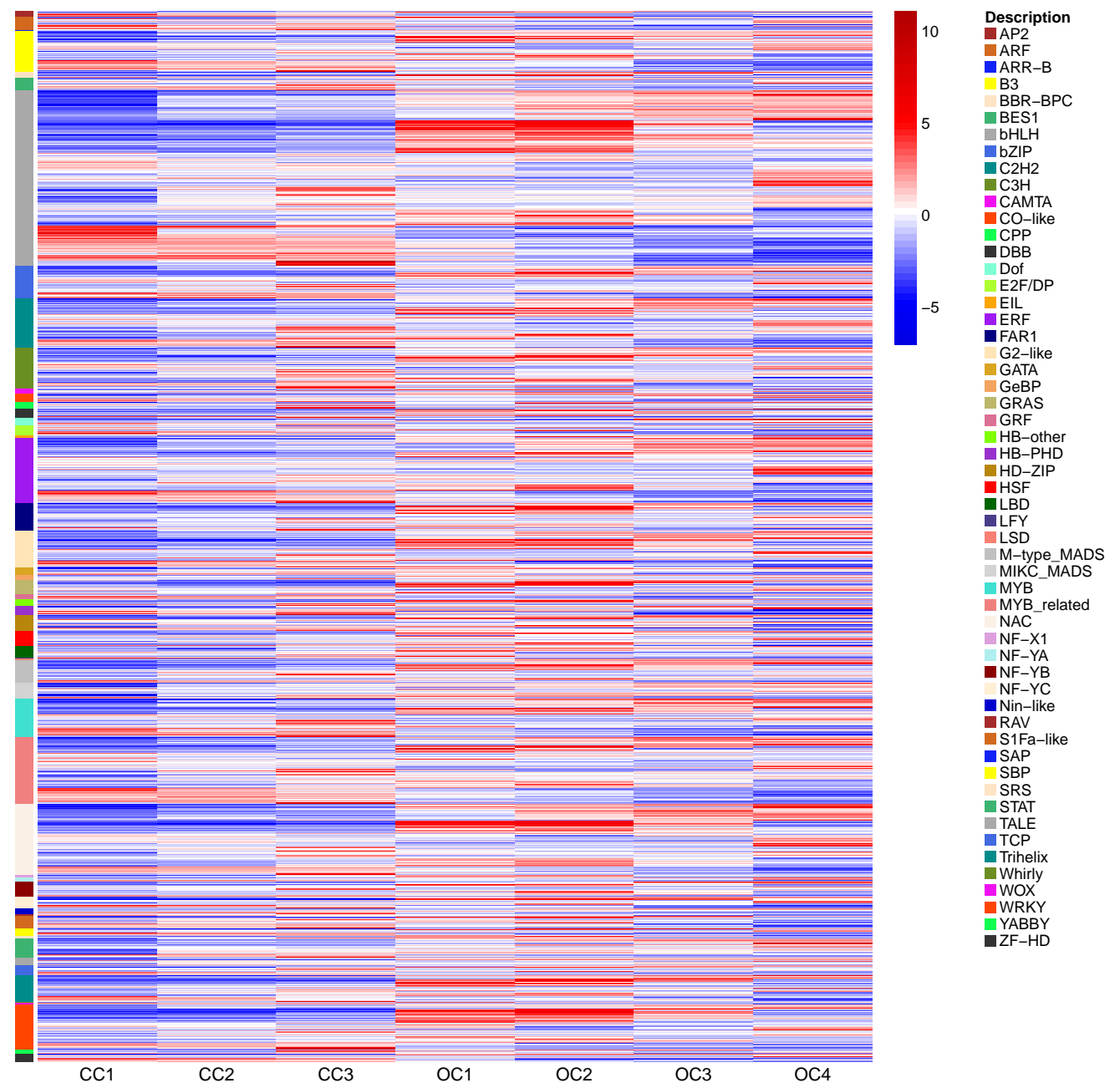

Supplement: Supplementary file 13 — Additional file 13: Figure S13. Expression levels of all the identified transcription families across the inflorescence development stages. [file 12864_2020_6670_MOESM13_ESM.pdf]

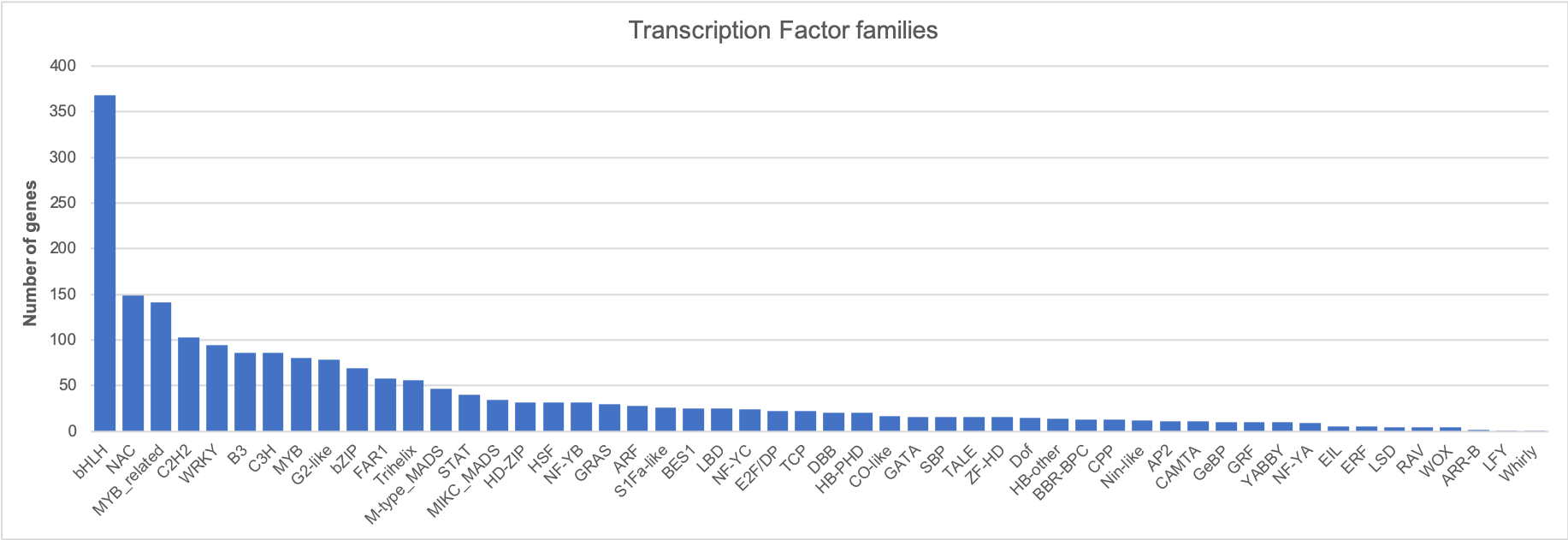

Supplement: Supplementary file 14 — Additional file 14: Figure S14. Number of genes for each differentially expressed transcription factor family across inflorescence development. [file 12864_2020_6670_MOESM14_ESM.png]
