# Supplementary material for: Hybrid transcriptome sequencing approach improved assembly and gene annotation in Cynara cardunculus (L.)
Source: BMC Genomics. 2020 Aug 21;21:317. doi: 10.1186/s12864-020-6670-5 (PMC7441626; doi:10.1186/s12864-020-6670-5)
Supplement: Supplementary file 15 — Additional file 15: Table S1. Primer sequences used for validation of RNA-seq data using qRT-PCR reactions. [file 12864_2020_6670_MOESM15_ESM.docx]

**Table S1.** Primer sequences used for validation of RNA-seq data using qRT-PCR reactions.

| **Gene name** | **Transcript name** | **Forward primer** | **Reverse primer** |
| --- | --- | --- | --- |
| *Actin* | Ccrd_v2_01732_g01-mRNA-1 | GTCGTACAACCGGTATTGTG | AGATCAAGACGGAGGATGG |
| *Chalcone isomerase (CHI)* | Ccrd_v2_05881_g03-mRNA-1 | GCAGGTGTGAGAGGAATG | CTTCCACTTACCGGCGA |
| *Chalcone synthase (CHS)* | Ccrd_v2_23309_g16-mRNA-1 | GCACGCTTGTGCAAAACAAC | TGCGTAATAGTTGGTGAGCC |
| *Costunolide synthase (COS)* | Ccrd_v2_23140_g16-mRNA-1 | GCCTTGTTTTTGGATGTGTT | ATTTCAGCTTGTACCTTGCT |
| *Flavonol synthase (FLS)* | Ccrd_v2_11699_g07-mRNA-1 | GGATGGGGAGGCACAATC | CTGAGATCGATCACCGGAA |
| *Germacrene A hydroxylase (GAO)* | Ccrd_v2_11621_g07-mRNA-1 | TGATTGGTACAATGCCACAT | CATTTCGGAGACGATACCAC |
| *MYB308-like* | Ccrd_v2_10696_g06-mRNA-1 | ACGCCGGATTGCAAAGA | TGAGATCTTCTTCTTGTTGTGA |
| *MYB86-like* | Ccrd_v2_18476_g12-mRNA-1 | CTTGGCAACAAGTGGTCTTT | AAAGCTTTCTTCTGATATGGGT |
