# Supplementary material for: Hybrid transcriptome sequencing approach improved assembly and gene annotation in Cynara cardunculus (L.)
Source: BMC Genomics. 2020 Aug 21;21:317. doi: 10.1186/s12864-020-6670-5 (PMC7441626; doi:10.1186/s12864-020-6670-5)
Supplement: Supplementary file 16 — Additional file 16: Table S2. RNA-seq data obtained with Illumina (SR-seq) and ONT (LR-seq) platforms. [file 12864_2020_6670_MOESM16_ESM.docx]

**Table S1**. RNA-seq data obtained with Illumina (SR-seq) and ONT (LR-seq) platforms. To assess reads quality they were aligned to reference genome.

|  | **SR-seq** | **LR-seq** |
| --- | --- | --- |
| Total reads sequenced | 1,023,768,646 | 1,445,444 |
| Average length, bp | 124 | 670 |
| Average insert size, bp | 225 | — |
| Reads longer than 500 bp | — | 871,590 (60%) |
| Transcript full-length reads | — | 1,346,930 (93%) |
| Maximal length, bp | 125 | 8,998 |
| Reads aligned to the reference genome | 906,739,878 (89%) | 1,246,785 (86%) |
| Remain after trimming  (used in the assembly) | 915,223,072 (89%) | 1,445,444 (100%) |
| Reads aligned to the assembly graph (BWA-MEM) | — | 1,364,987 (94%) |
|  |  |  |
