# Supplementary material for: Hybrid transcriptome sequencing approach improved assembly and gene annotation in Cynara cardunculus (L.)
Source: BMC Genomics. 2020 Aug 21;21:317. doi: 10.1186/s12864-020-6670-5 (PMC7441626; doi:10.1186/s12864-020-6670-5)
Supplement: Supplementary file 17 — Additional file 17: Table S3. Comparison of annotations of SR-seq and Hybrid-seq assemblies refined and updated by PASA software. [file 12864_2020_6670_MOESM17_ESM.docx]

**Table S2.** Genes count of different type of alternative splicing detected with SR-seq or Hybrid-seq by PASA analysis.

|  | **SR-seq** | **Hybrid-seq** |
| --- | --- | --- |
| Counts involved in alternative splicing | 12,975 | 13,155 |
| alternative acceptor | 3,265 | 3,392 |
| alternative donor | 2,747 | 2,846 |
| alternate exon | 1,756 | 1,912 |
| intron retention | 11,587 | 11,846 |
| exon skipping | 2,217 | 2,185 |
|  |  |  |
