# Supplementary material for: Hybrid transcriptome sequencing approach improved assembly and gene annotation in Cynara cardunculus (L.)
Source: BMC Genomics. 2020 Aug 21;21:317. doi: 10.1186/s12864-020-6670-5 (PMC7441626; doi:10.1186/s12864-020-6670-5)
Supplement: Supplementary file 18 — Additional file 18: Table S4. Functional annotation comparison of the transcriptome obtained using hybrid seq and SR-seq. [file 12864_2020_6670_MOESM18_ESM.docx]

**Table S3.** Functional annotation comparison of the transcriptome obtained using Hybrid seq and SR-seq.

|  |  | **Hybrid-seq** | **SR-seq** | | |
| --- | --- | --- | --- | --- | --- |
| Gene | | 35,956 | 34,268 |  |  |
| Annotated Genes | | 25,463 | 24,146 |  |  |
| GO Terms | Biological process | 15,783 | 15,059 | |  |
|  | Cellular component | 10,628 | 10,253 | |  |
|  | Molecular function | 20,778 | 19,628 | |  |
|  | Total | 47,189 | 44,940 | |  |
