# Supplementary material for: Hybrid transcriptome sequencing approach improved assembly and gene annotation in Cynara cardunculus (L.)
Source: BMC Genomics. 2020 Aug 21;21:317. doi: 10.1186/s12864-020-6670-5 (PMC7441626; doi:10.1186/s12864-020-6670-5)
Supplement: Supplementary file 19 — Additional file 19: Table S5. BLASTX result of phenylpropanoids, flavonoids and sesquiterpene lactones key genes detected in this study. [file 12864_2020_6670_MOESM19_ESM.docx]

**Table S5.** BLASTX result of phenylpropanoids. flavonoids and sesquiterpenes lactone key genes detected in this study.

| **Gene name** | **Identified transcript name** | **Description** | **E-value** | **Per. Ident.** | **Hit Species** |
| --- | --- | --- | --- | --- | --- |
| CHS | Ccrd_v2_23309_g16-mRNA-1 | chalcone synthase | 0.0 | 95.16% | Cynara cardunculus var. scolymus |
| CHI | split_gene_Ccrd_v2_05881_g03-Ccrd_v2_05881_g03-mRNA-1-m268 | chalcone isomerase | 4e-162 | 100.00% | Cynara cardunculus var. scolymus |
|  | Ccrd_v2_05881_g03-mRNA-1 | chalcone isomerase | 1e-156 | 100.00% | Cynara cardunculus var. scolymus |
| C3’H | Ccrd_v2_00602_g01-mRNA-1 | cytochrome P450 77A2-like | 0.0 | 100.00% | Cynara cardunculus var. scolymus |
|  | Ccrd_v2_00603_g01-mRNA-1 | cytochrome P450 77A2-like | 2e-48 | 100.00% | Cynara cardunculus var. scolymus |
| HCT | Ccrd_v2_24508_g17-mRNA-1 | shikimate O-hydroxycinnamoyltransferase-like | 0.0 | 95.94% | Cynara cardunculus var. scolymus |
|  | Ccrd_v2_12214_g08-mRNA-1 | shikimate O-hydroxycinnamoyltransferase-like | 0.0 | 100.00% | Cynara cardunculus var. scolymus |
| HQT | Ccrd_v2_03524_g02-mRNA-1 | hydroxycinnamoyl CoA quinate transferase 2 | 0.0 | 98.84% | Cynara cardunculus var. scolymus |
| COMT | Ccrd_v2_18337_g12-mRNA-1 | caffeic acid 3−O−methyltransferase | 0.0 | 91.27% | Cynara cardunculus var. scolymus |
|  | Ccrd_v2_20142_g13-mRNA-1 | caffeic acid 3−O−methyltransferase | 0.0 | 100.00% | Cynara cardunculus var. scolymus |
| 4CL | Ccrd_v2_10603_g06-mRNA-1 | 4-coumarate-CoA ligase | 0.0 | 100.00% | Cynara cardunculus var. scolymus |
|  | Ccrd_v2_10603_g06-mRNA-1.1.5b860188 | 4-coumarate-CoA ligase the | 0.0 | 99.02% | Cynara cardunculus var. scolymus |
|  | Ccrd_v2_10603_g06-mRNA-1.2.5b860188 | 4-coumarate-CoA ligase the | 0.0 | 94.79% | Cynara cardunculus var. scolymus |
| F3H | Ccrd_v2_09297_g05-mRNA-1 | naringenin 3-dioxygenase | 0.0 | 100.00% | Cynara cardunculus var. scolymus |
|  | Ccrd_v2_09297_g05-mRNA-1.1.5b860155 | naringenin 3-dioxygenase | 0.0 | 100.00% | Cynara cardunculus var. scolymus |
| FLS | Ccrd_v2_11699_g07-mRNA-1 | flavonol synthase | 4e-148 | 100.00% | Cynara cardunculus var. scolymus |
|  | Ccrd_v2_11708_g07-mRNA-1 | flavonol synthase | 7-92 | 100.00% | Cynara cardunculus var. scolymus |
| MYB12-like | Ccrd_v2_16361_g11-mRNA-1 | Transcription factor MYB12-like | 0.0 | 100.00% | Cynara cardunculus var. scolymus |
| MYB308-like | Ccrd_v2_18476_g12-mRNA-1 | Myb-related transcription factor MYB308-like | 0.0 | 100.00% | Cynara cardunculus var. scolymus |
| MYB86-like | Ccrd_v2_10696_g06-mRNA-1 | Transcription factor MYB86-like | 0.0 | 100.00% | Cynara cardunculus var. scolymus |
| GAS | Ccrd_v2_14245_g10-mRNA-1 | Germacrene A synthase short-form like | 0.0 | 96.68% | Cynara cardunculus var. scolymus |
|  | Ccrd_v2_11622_g07-mRNA-1 | Germacrene A synthase short-form like | 0.0 | 100.00% | Cynara cardunculus var. scolymus |
|  | Ccrd_v2_11622_g07-mRNA-1.1.5b8601e7 | Germacrene A synthase short-form like | 0.0 | 100.00% | Cynara cardunculus var. scolymus |
|  | Ccrd_v2_11622_g07-mRNA-1.2.5b8601e7 | Germacrene A synthase short-form like | 0.0 | 100.00% | Cynara cardunculus var. scolymus |
| GAO | Ccrd_v2_11621_g07-mRNA-1 | germacrene A oxidase | 0.0 | 100.00% | Cynara cardunculus var. scolymus |
|  | Ccrd_v2_11623_g07-mRNA-1 | germacrene A oxidase | 0.0 | 99.77% | Cynara cardunculus var. scolymus |
| COS | Ccrd_v2_23140_g16-mRNA-1 | costunolide synthase | 0.0 | 100.00% | Cynara cardunculus var. scolymus |
|  | Ccrd_v2_20636_g14-mRNA-1 | costunolide synthase | 0.0 | 100.00% | Cynara cardunculus var. scolymus |
| GDS | Ccrd_v2_13529_g09-mRNA-1 | (-)-germacrene D synthase | 0.0 | 99.52% | Cynara cardunculus var. scolymus |
|  | Ccrd_v2_22023_g15-mRNA-1 | (-)-germacrene D synthase | 0.0 | 94.36% | Cynara cardunculus var. scolymus |
